# Metformin plus lrinotecan in patients with refractory colorectal cancer: a phase 2 clinical trial

**DOI:** 10.1038/s41416-020-01208-6

**Published:** 2021-01-04

**Authors:** Arinilda Campos Bragagnoli, Raphael L. C. Araujo, Mauricio Wagner Ferraz, Lucas Vieira dos Santos, Kathia Cristina Abdalla, Fabiana Comar, Florinda Almeida Santos, Marco Antonio Oliveira, José Barreto Campello Carvalheira, Flávio Mavigner Cárcano, João Paulo da Silveira Nogueira Lima

**Affiliations:** 1 Hospital de Câncer de Barretos, Barretos, SP Brazil; 2 Universidade Federal de São Paulo, São Paulo, SP Brazil; 3 Hospital Israelita Albert Einstein, São Paulo, SP Brazil; 4 Beneficência Portuguesa de São Paulo, São Paulo, SP Brazil; 5 Fundação Faculdade Regional de Medicina de São José do Rio Preto, São José do Rio Preto, SP Brazil; 6 Aliança Instituto de Oncologia, Brasília, DF Brazil; 7 Universidade Estadual de Campinas, Campinas, SP Brazil; 8 AC Camargo Cancer Center, São Paulo, SP Brazil

**Keywords:** Drug development, Chemotherapy

## Abstract

**Background:**

Patients with refractory colorectal (CRC) cancer have few treatment options. This trial tests the combination of metformin and irinotecan in this setting.

**Methods:**

A phase 2 single-arm trial was conducted, patients received metformin 2500 mg orally a day plus irinotecan 125 mg/m^2^ intravenously weekly D1 and D8 every 21 days. The primary endpoint was the disease control rate according to the Response Evaluation Criteria in Solid Tumors version 1.1 at 12 weeks.

**Results:**

Between December 2015 and January 2018, 41 patients were enrolled. Seventeen patients (41%) met the primary endpoint of disease control in 12 weeks; hence, the study was deemed positive. The median progression-free survival was 3.3 months (CI 95%, 2.0–4.5 months), and the median overall survival was 8.4 months (CI 95%, 5.9–10.8 months). Both mutation RAS status and disease control at 12 weeks impacted overall survival in the multivariate model (HR 2.28, CI 95%, 1.12–4.7, *p* = 0.02; and HR 0.21, CI 95%, 0.08–0.5, *p* = 0.001, respectively). The most common adverse event was diarrhoea (29.2% grade 3).

**Conclusions:**

In this trial, metformin plus irinotecan demonstrated disease control in patients with refractory CRC. Further trials with optimised diarrhoea control are needed to confirm these results.

## Background

Over 1.8 million new colorectal cancer (CRC) cases were estimated to occur in 2018 worldwide.^1^ Colorectal cancer ranks third in global incidence but second in mortality, claiming 90,000 lives yearly.^1^ The treatment of patients with advanced metastatic colorectal cancer (mCRC) includes oxaliplatin and irinotecan-containing chemotherapy, and patients with RAS wild-type tumours should also receive anti-EGFR antibodies (e.g. cetuximab or panitumumab).^2^ New drugs have been developed in recent years for patients who are refractory to these agents. In the CORRECT trial, patients who received regorafenib, a multikinase targeted drug, had better progression-free survival (PFS) and overall survival (OS) than those who received placebo.^3^ Similar results were also found in the RECOURSE trial with trifluridine-tipiracil (TAS-102), a nucleoside analogue combined with a thymidine phosphorylase inhibitor.^4^ Another recent phase 2 trial showed a survival benefit with the combination of TAS-102 and bevacizumab, an anti-VEGF antibody.^5^

A recent meta-analysis showed that metformin, an oral biguanide hypoglycaemic drug, increases OS in diabetic patients with colorectal cancer likely due to its antitumour properties.^6^ Metformin emerges as an interesting therapeutic option for advanced heavily treated patients.^6–14^ Additionally, metformin is a low cost and widespread drug with known and easily manageable adverse events.

Despite the evidence of the anticancer effect of metformin, it has been based on few prospective studies, which only retrieved the role of this drug in cancer treatment.^15,16^ Moreover, the combination of metformin with irinotecan has not been explored in a clinical trial before. Thus, the objective of this study was to evaluate the efficacy and safety of irinotecan plus metformin in patients with refractory colorectal cancer. The primary endpoint was the disease control rate at 12 weeks, and the secondary endpoints were OS, PFS, toxicity and quality of life.

## Methods

### Patients

Eligible patients were adults over 18 years old with an Eastern Cooperative Oncology Group performance status of 0, 1 or 2 and with histologically confirmed colorectal adenocarcinoma. They must have evaluable metastatic CRC, previous treatment with fluoropyrimidine, irinotecan, oxaliplatin, and any approved antiepidermal growth factor receptor (EGFR) antibody if the tumour was RAS wild-type, and confirmed disease progression on these therapies. Patients must have adequate laboratory findings (haemoglobin > 9.0 g/dL, platelets > 100.000/mm^3^, neutrophil count > 1500/mm3, bilirubin level <1.5 times the upper limit of normal [ULN], aspartate aminotransferase and alanine aminotransferase <2. 5 times ULN or <5 ULN in case of liver metastasis, and creatinine <1.5 times ULN). The exclusion criteria were history of hypersensitivity to metformin, or chronic use of immunosuppressant drugs or corticosteroids (greater than 10 mg/day of prednisone). Patients with known brain metastasis, other malignancies, acquired immunodeficiency syndrome (AIDS), or active bleeding and pregnant or breastfeeding women were also excluded. Diabetic patients were accepted if they were not using metformin.

The study protocol (Supplement 1A—Study protocol) was approved by the institutional review board according to the ethics committee, and all participants provided written informed consent before any trial procedure.

### Study design and treatments

This study was an open-label, single-arm, phase 2 clinical trial conducted at Barretos Cancer Hospital in Brazil between December 2015 and January 2018. The primary endpoint was the disease control rate (DCR) at 12 weeks after the start of treatment. DCR was defined as stable disease, partial response, or complete response according to the Response Evaluation Criteria in Solid Tumors 1.1.^17^ The secondary endpoints were PFS, OS, toxicity and quality of life.

The study regimen consisted of metformin 2500 mg orally a day continuously plus irinotecan 125 mg/m2 intravenously weekly D1 and D8 every 21 days until disease progression, unacceptable toxicity, or withdrawal of consent. Every cycle had duration of 21 days. In cases of grade 3 or intolerable grade 2 adverse events, dose reduction to 1500 mg/day of metformin and/or 20% reduction of irinotecan were recommended.

The initial dose of metformin was 1500 mg/day, and after 7 days, the patients were reassessed for adherence and tolerability. If no grade 3 events occurred, the dose was increased to 2500 mg/day. The subsequent visits were on day 1 of every cycle. Toxicities were classified according to the National Cancer Institute Common Toxicity Criteria for Adverse Events version 4.0. Radiographic evaluations with computerised tomographic scans of the thorax, abdomen and pelvis were performed on weeks 6 and 12 after starting treatment and then every 12 weeks. To assess quality of life, the EORTC QLQ-C30 questionnaire^18^ was administered to all patients in the first treatment visit and on weeks 3, 6 and 9 and then every four cycles of chemotherapy.

### Statistical analysis

Sample size was calculated according to the optimal design of Simon for phase 2 clinical trials.^19^ Considering a DCR of 13.2% on week 12 with placebo as the third line,^3^ we believe that it would be clinically significant to double this DCR to 26.7%. Considering an alpha error of 10% and a beta error of 20%, we planned to enroll 21 patients in the first phase. If at least four patients met the primary endpoint, the accrual was expanded to a total of 41 patients. This study would be considered positive if at least nine patients out 41 had disease control on week 12.

The dichotomous outcomes of efficacy, as well as the baseline characteristics of patients and adverse events, were analysed using Fisher’s exact test or Χ² test. PFS was calculated as the time interval between treatment start until the date of radiological progression (RECIST 1.1) or death, and OS was calculated as the interval between the first day of treatment and the date of death. Both were estimated using the Kaplan–Meier method. A *p* value of <0.05 was considered significant for all analyses. Only variables that presented *p* < 0.2 in the univariate analysis were included in the multivariate analysis of overall survival. For quality of life analysis, the mean scores on each scale of the EORTC QLQ-C30 questionnaire were compared at the beginning and end of the study treatment using the Wilcoxon signed-rank test. The other results were reported using descriptive statistics. SPSS software version 21.0 (SPSS Inc., Chicago, IL, United States) was used for the statistical analysis.

## Results

**Fig. 1:**
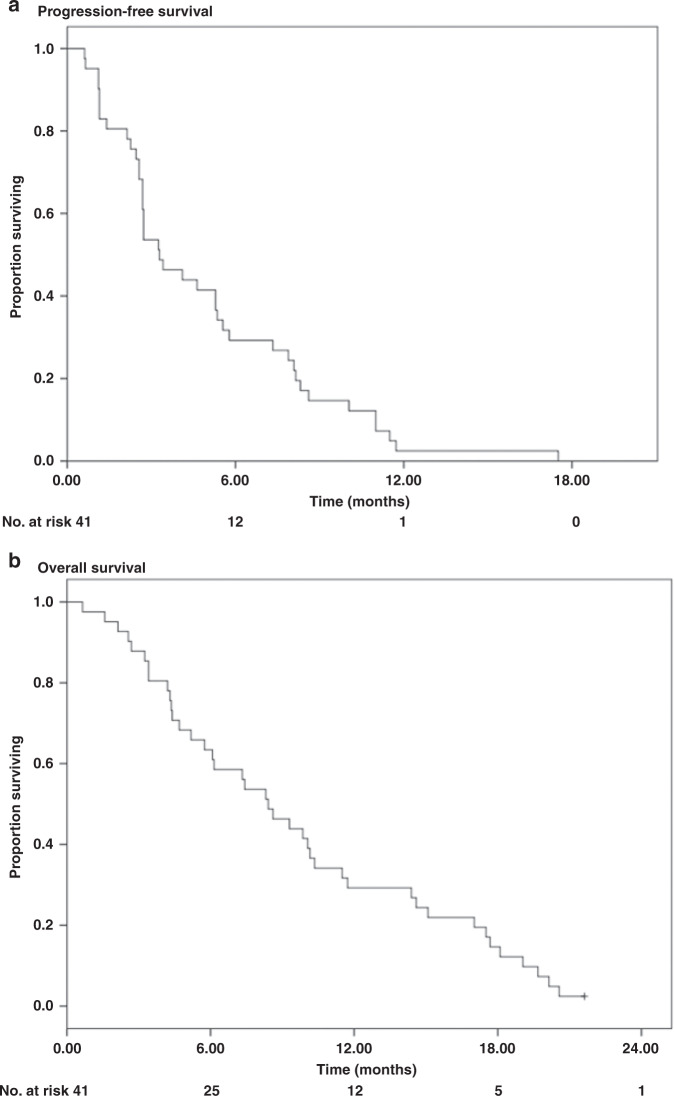
Kaplan–Meier curve of progression-free survival and overall survival for the entire study population. **a** Progression-free survival; **b** overall survival.

From December 2015 to January 2018, 48 patients consented to participate in this study. Five patients were excluded because of screening failure (four abnormal laboratory findings and one early cancer-related death) and two withdrew consent. A total of 41 patients were included in the intention-to-treat population, as demonstrated in Fig. 1. The demographic characteristics, baseline disease characteristics and chemotherapy performed before study initiation are shown in Table 1. In brief, the median age was 55 years (range, 22–78 years), 27 patients (66%) had a RAS mutation, and 14 patients (34%) had three or more sites of metastatic disease. Only 11 patients (27%) presented a body mass index equal to or higher than 30 kg/m2. All patients received at least two lines of chemotherapy, with 24 patients (58.5%) undergoing three or more lines before starting this clinical trial. The chemotherapy most often used in the first line of treatment was based on oxaliplatin. Only 29% of patients received bevacizumab combined with any line of chemotherapy prior to inclusion in the research study.

**Table 1:** Demographic, baseline disease characteristics and chemotherapy performed before study enter (*N* = 41).

Characteristic	Number (%)
Age, median (range), years	55 (22–78)
Sex	
Male	26 (63)
Female	15 (37)
ECOG performance status^a^	
0	6 (15)
≥1	35 (85)
Location of primary tumour	
Left colon	21 (51)
Right colon	20 (49)
RAS mutation status^b^	
Wild-type	14 (34)
Mutated	27 (66)
Obesity (BMI ≥ 30 kg/m^2^)^c^	
Yes	11 (27)
No	30 (73)
Number of metastatic sites	
1	8 (20)
2	19 (46)
≥3	14 (34)
Time since diagnosis of metastatic disease	
<18 months	19 (46)
≥18 months	22 (54)
First-line chemotherapy (*N* = 41)	
Oxaliplatin based^d^	30 (73)
Irinotecan based^e^	10 (25)
5-FU	1 (2)
Second-line chemotherapy (*N* = 41)	
Oxaliplatin based	14 (34)
Irinotecan based	27 (66)
Third-Line Chemotherapy (*N* = 24)	
Oxaliplatin based	8 (33)
Irinotecan based	9 (38)
Cetuximab + irinotecan	7 (29)
Fourth-line chemotherapy (*N* = 7)	
Oxaliplatin based	1 (14)
Cetuximab + irinotecan	5 (72)
Cetuximab	1 (14)
Fifth-line chemotherapy (*N* = 1)	
Oxaliplatin based	1 (100)
Addition of bevacizumab (any line of chemotherapy)	
Yes	12 (29)
No	29 (71)

median (range).

^a^Eastern Cooperative Oncology Group.

^b^Rat sarcoma virus.

^c^Body Mass Index.

^d^Oxaliplatin-based chemotherapy: oxaliplatin, leucovorin and bolus 5-FU (FLOX) or infusional 5-FU (FOLFOX) or oxaliplatin plus capecitabine (CAPOX).

^e^Irinotecan, leucovorin and bolus 5-FU (IFL) or infusional 5-FU (FOLFIRI).

The median time to progression after the first exposure to irinotecan combined with 5-fluorouracil (IFL or FOLFIRI regimens) was 6.2 months (range, 2.1–20.7 months), and the median time between the last exposure to irinotecan and the start of study treatment was 2.6 months (range, 0.49–45.77 months). Almost two-thirds of patients (63%, 26 patients) had received irinotecan-based chemotherapy immediately before entering the study.

**Fig. 2:**
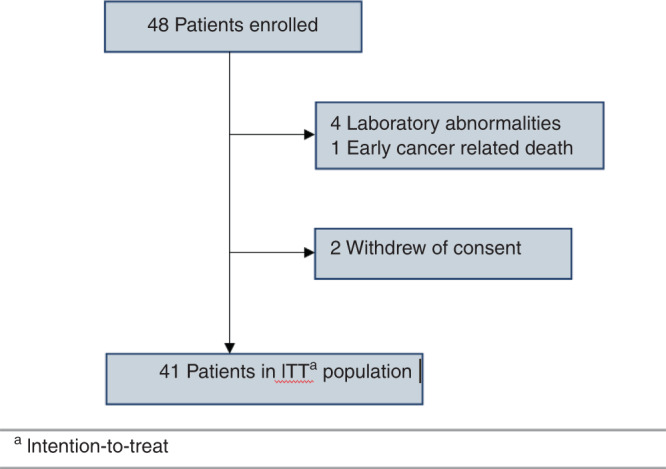
CONSORT diagram. ITT intetion to treat.

At the time of data cut-off in January 2020, the median follow-up for living patients was 8.2 months (range, 0.9–21.4 months), and all 41 patients had already progressed. None of the patients had an objective response (complete or partial response by RECIST 1.1 radiological evaluation); however, in the first period of radiological evaluation in the 6th week of treatment, 32 patients maintained stable disease, with a disease control rate of 78% (95% CI, 62–89%). The study met its primary endpoint, with a DCR at 12 weeks in 17 out 41 patients (41% DCR, 95% CI, 26–58%). The median progression-free survival was 3.3 months (95% CI, 2.02–4.55 months), and the median overall survival was 8.4 months (95% CI, 5.93–10.88 months), as shown in Fig. 2a, b.

We also confirmed that 12-week DCR, the primary outcome of the study, was correlated with PFS (*p* = 0.009) and OS (*p* = 0.001). The median OS of patients who achieved 12-week DCR was 11.5 months (95% CI, 5.4–17.6 months), compared to 4.4 months (95% CI, 3.3–5.5 months) for those who did not obtain disease control in 12 weeks (*p* = 0.001).

On univariate analysis, we analysed possible associations between clinical and demographic variables and survival outcomes. The 12-week DCR was correlated with time to progression on first pre-trial irinotecan-based chemotherapy, namely the longer time to progression on previous irinotecan-therapy, the higher likelihood of 12-week DCR (*p* = 0.001). Older patients, above the 55-year median age fared better than younger ones, with longer OS (10.1 months for median OS for older versus 4.7 months for median OS for younger patients; *p* = 0.02). There was also a trend toward increased overall survival in patients with wild-type RAS (*p* = 0.06) (Table 2).

**Table 2:** Univariate and multivariate analysis for predictor in overall survival.

	Univariate analysis		Multivariate analysis	
Characteristic	Median OS (95% CI), months	*P* value	Hazard Ratio^a^ (95% CI)	*P* value
Age				
<55 years	4.7 (2.9–6.5)	0.02	Reference	0.54
≥55 years	10.1 (7.6–12.5)		0.58 (0.27–1.07)	
Sex				
Male	8.4 (5.1–11.7)	0.88		
Female	7.3 (1.9–12.7)			
Primary tumour location				
Right colon	8.6 (4.9–12.25)	0.43		
Left colon	8.3 (4.3–12.3)			
RAS status				
Wild-type	11.5 (3.7–19.2)	0.06	Reference	0.02
Mutated	5.2 (2.9–7.5)		2.28 (1.12–4.7)	
BMI ≥ 30 kg/m^2^				
No	7.4 (3.2–11.7)	0.72		
Yes	8.4 (.1–11.8)			
Number of chemotherapy lines				
2	5.7 (3.1–7.7)	0.25		
3	9.8 (5.6–14.1)			
≥4	10.1 (8.1–12)			
Use of bevacizumab?				
No	10 (7.3–12.8)	0.20		
Yes	6.1 (2.35–9.9)			
Time to progression on first irinotecan-based chemotherapy				
<6,2 months	5,2 (2.2–8.2)	0.087	1.37 (0.6–3.1)	0.437
≥6,2 months	10.1 (7.45–12.7)		Reference	
Time since diagnosis of metastatic disease				
<18 months	5.7 (3.7–7.8)	0.32		
≥18 months	9.3 (7.3–11.3)			
Irinotecan-free interval between last exposure and treatment start				
<2.63 months	6.1 (2.6–9.5)	0.96		
≥2.63 months	9.9 (7.4–12.3)			
Disease control in week 12th				
Yes	11.5 (5.4–17)	0.001	0.21 (0.08–0.5)	0.001
No	4.4 (3.3–5.5)		Reference	

The univariate analysis for PFS is described on Table 3, multivariate analysis was not performed because there were no statistically significant findings on univariate analysis. Supplement 1B—Progression Free survival and Overall Survival according to irinotecan refractoriness shows the survival and progression-free survival in patients with irinotecan-free interval inferior and superior to 3 months.

**Table 3:** Univariate analysis for predictor in progression-free survival.

	Univariate analysis	
Characteristic	Median PFS (95% CI), months	*P* value
Age		
<55 years	2.7 (2.4–2.9)	0.58
≥55 years	5.3 (3.3–7.2)	
Sex		
Male	2.7 (0.14–5.3)	0.65
Female	3.2 (1.5–5.0)	
Primary tumour location		
Right colon	4.6 (2.9–6.3)	0.33
Left colon	2.7 (2.0–3.3)	
RAS status		
Wild-type	5.3 (0–11.2)	0.09
Mutated	2.7 (2.0–3.4)	
BMI ≥ 30 kg/m^2^		
No	3.2 (1.2–5.3)	0.47
Yes	3.2 (1.7–4.7)	
Number of chemotherapy lines		
2	2.7 (2.1–3.3)	0.72
3	5.3 (1.5–9.0)	
≥4	4.1 (0.5–7.6)	
Use of bevacizumab?		
No	3.4 (1.9–4.9)	0.33
Yes	2.6 (1.7–3.7)	
Time to progression on first irinotecan-based chemotherapy		
<6,2 months	2.6(2.5–2.8)	0.23
≥6,2 months	5.7 (2.7–8.8)	
Time since diagnosis of metastatic disease		
<18 months	3.2 (2.6–3.8)	0.40
≥18 months	3.4 (1.25–5.5)	
Irinotecan refractoriness		
Yes	2.6 (2.5–2.9)	0.15

95% CI: Two-sided 95% exact confidence interval. BMI: body mass index.

In the multivariate analysis of OS, both disease control at 12 weeks and the presence of mutated RAS were independent prognostic factors, increasing (HR 0.21; 95% CI 0.08–0.5; *p* = 0.001) and worsening OS (HR = 2.28; 95% CI 1.12–4.7; *p* = 0.023), respectively, as shown in Table 2.

Common adverse events were grade 1 and 2 nausea, vomiting, asthenia and diarrhoea, this last being the commonest one. No patient developed blood glucose abnormalities (hyperglycaemia or hypoglycaemia) due to treatment and there was no unexpected toxicity among diabetic patients.

The most frequent severe (grade 3 or 4) event was diarrhoea, with almost 30% of cases being grade 3; still, no patient required hospitalisation to control this symptom. The total number of serious adverse events was 10, with most of them related to disease progression (7 events), two febrile neutropenia and one death of unknown cause. There was no death due to toxicity. Table 4 depicts the distribution and frequency of adverse events.

**Table 4:** Adverse events.

Adverse event	Grade 1 -2	Grade 3
Diarrhea	26 (63%)	12 (29%)
Nausea	21 (51%)	1 (2%)
Vomit	19 (46%)	1 (2%)
Oral Mucositis	4 (10%)	1 (2%)
Fatigue	21 (51%)	6 (15%)
Neutropenia	7 (17%)	3 (7%)
Febrile Neutropenia	—	2 (5%)
Anemia	1 (2%)	1 (2%)
Total number of serious adverse events	10	

Number between parentheses represent percent values.

A total of 217 cycles were administered, with a median of four cycles per patient (1–17 cycles). The treatment was delayed because of an adverse event in 10 cycles (4.6%) and administered in a reduced dose in 90 cycles (41%). The mean (standard deviation) relative dose intensities were 87% (16%) for metformin and 90% (10%) for irinotecan. Unfortunately, pharmacokinetic analysis was not performed.

The EORTC QLQ-C30 questionnaire was used to measure quality of life. The scores could range from 0 to 100, with higher scores representing high functioning on the global health scale and functional scale while higher scores on symptom scales indicate their worsening. In comparing the baseline and end of treatment scores, we found no significant differences in the global health score and functional scales. However, assessing the variables individually, we found worsening in some symptoms: fatigue (*p* = 0.005), diarrhoea (*p* = 0.01), nausea (*p* < 0.001) and loss of appetite (*p* = 0.01).

After discontinuation of irinotecan plus metformin only three patients received another chemotherapy treatment (all of them received an oxaliplatin-based chemotherapy).

## Discussion

This was the first clinical trial that assessed the combination of irinotecan and metformin for heavily treated metastatic colorectal cancer patients. This study met its primary endpoint, with 41% of patients experiencing disease control in 12 weeks. Furthermore, the 12-week disease control posed as a good marker for PFS and OS, as patients with disease controlled at this timeframe had prolonged survival. Recognising the limitations of a phase 2 single-arm trial but comparing the results with those of phase 3 trials in the setting of refractory colorectal cancer, we found a similar overall survival (8.4 months) rates compared to regorafenib in the CORRECT trial (6.4 months)^3^ and TAS-102 in the RECOURSE trial (7.1 months).^4^

The potential mechanisms responsible for the antitumour effects of metformin remain unclear. An indirect effect (insulin dependent) was hypothesised in which insulin acts as a growth factor.^20,21^ Through a direct effect (insulin-independent), metformin causes an inhibition of the mitochondrial respiratory chain on tumour cells that increases the AMP active protein kinase.^20,21^ This enzyme is responsible for multiple actions involved in protein synthesis and cell proliferation, such as the mammalian target of rapamycin (mTOR) pathway.^20,21^ Moreover, a study conducted by our team showed a synergistic effect of another mTOR inhibitor (rapamycin) in combination with irinotecan in mice with colorectal cancer treated with these agents.^22^

A possible alternative explanation for the positive results of this trial would be simply due to rechallenge of irinotecan. The rechallenge effect is defined as the readministration of drug to a patient who has developed resistance while being on treatment.^23^ For oxaliplatin, three retrospective series have reported response rates of up to 20% and disease stabilisation rates of more than 40% following reintroduction after prior discontinuation. However, rechallenge is usually an acceptable option if the reintroduction of the drug occurs within 6 months after the pause of that treatment.^24,25^ All patients in our study progressed during the use or up to 6 months after the interruption of irinotecan, with a median irinotecan-free interval of ~2 months. Thence, they all were irinotecan-refractory at trial enrolment. In addition, more than half of the patients were exposed to more than three lines of chemotherapy and received irinotecan as the last treatment regimen. These data demonstrate that our population has been extensively treated and has shown resistance to irinotecan, making unlikely that rechallenge alone would be responsible for the positive results.

As expected in this heavily treatment scenario, there was no objective response in the current trial, similar to studies with regorafenib, TAS-102 and the combination of TAS-102 and bevacizumab.^3–5^ A phase 1B trial showed an encouraging response rate of 33% in heavily pretreated patients with microsatellite-stable colorectal cancer who received regorafenib plus the checkpoint inhibitor nivolumab.^26^ However, a phase 3 trial is needed to confirm these initial positive results of immunotherapy-based treatment.

Another interesting result is the possible association between RAS mutation status and OS. In a preclinical study, it was suggested that metformin induces apoptosis and inhibits cell proliferation in cell models of endometrial cancer, especially in those with KRAS mutations.^27^ A recent analysis of a phase 2 clinical trial that evaluated the use of metformin associated with chemotherapy in advanced lung cancer showed that patients with mutated KRAS had better PFS and OS.^16^ However, in our study, patients with a RAS mutation had poorer OS than wild-type patients. KRAS mutation is considered a negative prognostic marker in metastatic CRC.^28^ Hence, we believe that the worse survival observed in patients with RAS mutations in our study is probably associated with the negative prognostic impact of this alteration and not with a predictive value of response to the combination of metformin and irinotecan; the absence of a control arm in our study, however, prevents us from reaching a definitive conclusion.

Most side effects from study treatment were mild (grade 1 or 2), apart from diarrhoea, which was grade 3 in almost 30% of patients, higher than regorafenib (7%) and TAS-102 (3%).^3,4,15^ This finding was expected with the combination of two drugs capable of increasing dejections. Despite this, none of the patients required a hospital stay for the symptomatic management of diarrhoea, and all patients obtained control with symptomatic drugs such as loperamide. A possible strategy to reduce this adverse event is the prophylactic use of antidiarrhoeal medications or lower dose throughout the study, as roughly 40% of patients were dose reduced during trial therapy. Other interesting strategy is the translational study to better select the patient that the treatment will be beneficial. A recent study showed that the rs2282143 single nucleotide polymorphism in breast cancer is related to a high sensitivity to metformin.^29^ In spite of diarrhoea, the irinotecan-metformin combination had negligible hypertension or hand foot syndrome, toxicities common with other available drugs for advanced colorectal cancer.^3^

Despite the adverse events that the patients presented in our study, there was a high adherence to the prescribed metformin dose. Compared to regorafenib, there was less cycle interruption due to toxicity (4.6% for metformin and irinotecan vs 61% for regorafenib) and a similar dose reduction (41% for metformin and irinotecan vs 38% for regorafenib).^3^

The quality of life measured with the EORTC QLQ-C30 questionnaire demonstrated a worsening regarding some symptoms associated with the combination of irinotecan and metformin, such as diarrhoea, fatigue, loss of appetite and nausea. Regorafenib was also associated with a decrease in quality of life scores, but there was no difference compared to placebo.^3^ Therapies for metastatic cancer are commonly associated with adverse events that can significantly reduce quality of life. A systematic review published recently showed that 55% of patients consider life time and quality of life equally important, and 18% consider life time more important.^30^ In a scenario of refractory colorectal cancer, the modest improvement in overall survival with chemotherapy is necessary to discuss with the patient as well as the possibility of worsening quality of life with treatment.

In the current landscape of multiple approved drugs for refractory colorectal cancer, such as regorafenib, trifluridine-tipiracil and immune checkpoint inhibitors, the combination of metformin and irinotecan is a low cost readily available option. Health costs are an important issue worldwide. A recent cost-effectiveness study showed that both regorafenib and TAS-102 are not cost-effective.^31^ Furthermore, regorafenib and TAS-102 are not available in several countries, including for patients in the public health system in Brazil. In this context, this study showed a possible less expensive option to treat patients with refractory colorectal cancer that is especially promising in developing countries.

## Limitations

This study has some limitations. It is a single-centre phase 2 trial that was not randomised. Most patients were not previously exposed to antiangiogenic agents, which was because our patients were from the public health system, which did not usually reimburse these high-cost drugs. Metformin has a possible antiangiogenic effect,^32^ because most of our patients were not previously exposed to bevacizumab our data could not be replicated to this population. Another limitation is that patients received anti-EGFR only in third line, this fact preclude rechallenge of this drug. In addition, the possibility of that the positive results of this trial were due to a rechallenge effect of irinotecan, the exclusive action of metformin, favourable tumour biology or even by chance cannot be excluded.

## Conclusion

The combination of metformin and irinotecan seems to be an effective regimen in patients with refractory colorectal cancer, demonstrating a good safety profile. However, randomised clinical trials are needed to confirm our results.

## Supplementary information

Supplement 1

## Data Availability

All data are available upon request.
