# Supplementary material for: Metformin plus lrinotecan in patients with refractory colorectal cancer: a phase 2 clinical trial
Source: Br J Cancer. 2021 Jan 4;124(6):1072–8. doi: 10.1038/s41416-020-01208-6 (PMC7961008; doi:10.1038/s41416-020-01208-6)

1. **Study Protocol**

Phase 2 single arm clinical trial evaluating the combination of metformin and irinotecan in the treatment of refractory colorectal cancer

Principal investigator:

Dr. Arinilda Campos Bragagnoli

Clinical Research Unit and Department of Clinical Oncology, Barretos Cancer Hospital, Barretos, São Paulo.

Sub-Investigators:

João Paulo da Silveira Nogueira Lima, PhD

Raphael L. C. Araújo, PhD

Kathia Cristina Abdalla,MD

Florinda Almeida Santos MD

Fabiana Comar, MD

Lucas Vieira dos Santos,MD

José Barreto Campello Carvalheira, PhD

July, 2013

Informative Summary

| Research Medication | Metformin |
| --- | --- |
| Study Phase | II |
| Title of the Study | Phase II clinical study, single arm, evaluating the combination of metformin and irinotecan in the treatment of metastatic colorectal cancer refractory to chemotherapy |
| Introduction | Annually, colorectal cancer affects approximately 1.25  million people worldwide. (1) Despite the undeniable  advances in early disease screening and curative  management, about 50% of patients have metastatic disease,  either at diagnosis or in its evolution, an incurable and fatal  condition in most cases. (1) As a result, 600,000 people die  each year despite therapeutic advances. Thus, it is essential  to increase and improve the therapeutic range of metastatic  colorectal cancer (mCRC) in order to provide better care for  this huge number of people. In Brazil, consolidated data show  that colon and rectal cancer is the third most common cancer,  being diagnosed in about 30,000 people a year.  Since the estimated number of individuals with type 2  diabetes taking metformin daily is over 100 million,  epidemiologists have examined the effect of metformin on  cancer incidence. Initial results showed a reduction in the  amount of cancer in individuals taking metformin compared to  any other drug. Similarly, a recent breast cancer study in  patients with type 2 diabetes who were taking metformin  showed a significant increase in pathological complete  response, and a large phase III study to evaluate metformin  as adjunctive treatment is under development.  The mechanism by which metformin exerts its effects  on antineoplastic therapy is unclear, however AMPK inhibition  of the mTOR pathway is one of the main mechanisms pointed  out.  Preclinical data from our laboratory showed synergistic  antitumor effect of irinotecan combined with metformin on  growth of graft colon cancer lineage in immunodeficient mice (SCID mice) following treatment of mice with irinotecan and rapamycin at doses that reduce tumor growth “in vivo” for each agent alone. Although tumor growth has been reduced in mice treated with each agent alone, the tumors still increased approximately fivefold. In contrast, tumor growth in animals treated with the combined drugs was completely blocked.  These data demonstrate that the combined use of AMPK  activator with irinotecan dramatically reduces tumor growth  “in vivo”.  Metformin is a recognized AMPK activator. |
| Purpose/ Justification | In this study we are planning to evaluate the safety and efficacy of metformin combined with irinotecan in patients with recurrent or metastatic colon and rectal cancer (mCRC) who have failed all conventional chemotherapy regimens available and are not candidates for surgical treatment with curative intent. These patients do not have valid therapeutic options approved by national regulatory agencies. |
|  | The treatment of cancer represents a public health challenge, so federal expenses are rising. Colorectal cancer appears in this scenario as one of the most incident neoplasms in Brazil and as a disease in which therapeutic alternatives are restricted after their recurrence. On the other hand, currently established therapies are considered fertile soil for the development of modern cancer drugs. The union between the use of drugs already established with a new indication results in less spending both in the experimental stage, not being necessary to establish a toxicity profile, and in the application phase, which can result in reduced costs, since these drugs no longer have patents. |
| Objectives | Primary Objectives: To evaluate the disease control rate at week 12 (after four treatment cycles) and the safety of the combination of metformin and irinotecan in the treatment of patients with refractory mCRC treated with chemotherapeutic drugs available in Brazil for this disease.  Secondary Objectives: To evaluate response duration, objective response, disease progression-free survival (DPS), and overall survival (OS). Still, quality of life will be evaluated (EORTC QLQ-C30 and QLQCR29 questionnaire). |
| Study Design | This is a multicentric, prospective, single arm, phase II, two-stage study to investigate the efficacy and safety of the combination of irinotecan and metformin in patients with metastatic colorectal cancer. If being a promising regimen, there may be a further extension to a phase III study. The primary endpoint is the disease control rate at the 12^th^ week of treatment. |
|  | The primary endpoint is the disease control rate (stable disease + partial response + complete response) at the twelfth week of treatment. The efficacy assessment will be by intention of treatment. The metformin and irinotecan combination will be administered continuously until disease progression by RECIST v1.1 criteria or in case of severe toxicity or withdrawal of consent.  Sample size was calculated according to the optimal design of Simon. Considering a DCR of 13,2% on week 12 with placebo in third line, we believe that it would be clinically significant to increase this DCR to 26,7% .Considering an alpha error of 10% and a beta error of 20%, we planned to enroll 41 patients. This study would be considered positive if at least 9 patients had disease control on week 12.  This is a multi-institutional study, and it will take place at the Clinical Research Unit of Barretos Cancer Hospital.  Metformin will be given at a dose of 1500mg / day orally for 7 days, initially, and escalated to 2500mg / day if tolerated after one week of use, plus irinotecan at a dose of 125 mg per square meter body surface area D1 and D8 every 21 days. All patients will be treated until disease progression or appearance of prohibitive toxic effects. Those with a complete radiological response (CR) may discontinue irinotecan use two cycles after the diagnosis of CR (only if a total of six cycles is reached) at the investigator’s discretion, as well as those without presenting progression after two consecutive response assessments.  Tumor response will be assessed by computerized tomography (CT) or magnetic resonance imaging (MRI) every two cycles in the combined metformin and irinotecan phase and every twelve weeks during the metformin phase alone.  Toxic effects will be analyzed according to the National Cancer Institute Common Toxicity Criteria for Adverse Events, version 4 (NCI CTCAE v4). |
| Population | The target population includes adult patients with histologically confirmed or recurrent metastatic RCC refractory to treatment with oxaliplatin, irinotecan, fluoropyrimidine and an anti-EGFr agent if it is wild K-ras. |
| Inclusion/  Exclusion  Criteria | Inclusion Criteria:  Patients aged 18 years and over;  Patients with colon and rectal adenocarcinoma, with  recurrent or metastatic disease, refractory to treatment  based on oxaliplatin, irinotecan, fluoropyrimidine and, in  the case of wild K-ras patients, should also be resistant to  cetuximab or panitumumab;  Evaluable disease according to RECIST 1.1 criteria;  ECOG 0-2;  Suitable hematological functions (hemoglobin ≥ 9.0 g / dL;  neutrophil count ≥ 1,500 / mm3 and platelet count ≥  100,000 / mm3), adequate renal function (serum creatinine  less than 1.5 times the normal upper limit) and adequate  liver function (bilirubin less than 1.5 times the normal limit;  aspartate aminotransferase and alanine aminotransferase  less than five times the normal limit when there is liver  metastasis, or 2.5 times the normal limit, when the liver is  free of secondary lesions);  Exclusion Criteria:  Patients with known hypersensitivity to and/or prior to  irinotecan and metformin therapy;  Chronic treatment with immunosuppressive agent or  corticosteroids at a dose greater than 10 mg/day of  prednisone ;  Patients with known central nervous system (CNS)  metastases;  Patients with a previous diagnosis of another neoplasm,  except for basal cell carcinoma of the skin and noninvasive  cervical cancer;  Known HIV-positive patients will be accepted provided they do not have active AIDS-defining illness or AIDS- associated condition that compromises the safety of patients with stable antiretroviral treatment and reconstituted immunity (CD4 lymphocyte count ≥200/mm³, regardless of viral load);  Chronic or acute active infection (except known HIV  infection with no relevant immune repercussions);  Patients with active bleeding or hemorrhagic diathesis;  Patients with any uncontrolled serious medical condition or  other conditions that may affect their participation in the  study;  Patients who are taking another study medication or who  have received study drugs for less than 4 weeks;  Patients who are pregnant or nursing;  Diabetic patients on metformin treatment;  Absolute contraindication to loperamide use. |

| Dose, treatment cycle | Metformin will be given at a dose of 1500mg/day (two pills in the morning at breakfast and one at dinner) for seven days. If there is no prohibitive adverse effect at 1500mg/day, the dose will be increased to 2500mg/day (three pills in the morning at breakfast and two at dinner), and this dose will be maintained until PD or unacceptable toxicity. Metformin will not be administered within two days prior to contrast infusion examinations at the discretion of the radiologist to minimize the risks of lactic acidosis.  Irinotecan will be administered at a dose of 125 mg/m² intravenously D1 and D8 every 21 days. |
| --- | --- |
| Preparation and administration | Metformin will have 500mg presentation. Not dosed by weight or body surface area. Irinotecan will be diluted in 5% dextrose solution. Its infusion will be performed intravenously, lasting 90 minutes. The irinotecan infusion will be preceded by the infusion of dexamethasone 20mg and ondansetrone 16mg. |
| Schedule of visits and evaluations | Study evaluations will be performed at the screening period (D-21 to D-1), the day of study initiation (D1), at D8 of the first cycle and at D1 of the subsequent cycles. |
| Effectiveness Assessments | Tumor assessment (CT or thoracic MRI, abdominal and pelvic) will be performed every 6 weeks until the 12^th^ week of initiation of treatment and after 12 weeks or at any time during the study where clinical progression is suspected. Any suspicion of progression due to clinical deterioration should be accompanied by radiological confirmation.  All images will be analyzed locally at the centers and this interpretation will be used for all clinical decisions. RECIST criteria will be used for primary endpoint analysis. |

1. Introduction

1.1 Colorectal cancer

  Annually, colorectal cancer affects approximately 1.25 million people worldwide. (1) In Brazil, consolidated data show that colon and rectal cancer is the third most common cancer, being diagnosed in about 30,000 people a year.

The importance of this disease is felt in the volume of studies that investigate its biology. Many of these studies had direct repercussions on their management. The oncogenesis of this disease is quite well described, which allowed the elaboration of effective strategies for screening the premalignant component and early disease, with direct repercussions on survival. (2-6)

Refinements of local care, through isolated surgery or combination of radiotherapy and surgery have improved local control of the disease. (7-9) Pre- and postoperative (neo- or adjuvant) chemotherapy has been used since the eighties, improving cure rates consistently. (10, 11) Despite undeniable breakthroughs in early disease screening and curative management, about 50% of patients present metastatic disease, either at diagnosis or disease progression, incurable condition and fatal in most cases. (1) Thus, 600,000 people die each year despite therapeutic advances. Therefore, it is essential to increase and improve the therapeutic range of metastatic colorectal cancer (mCRC) in order to provide better care for this great amount of people.

Even considering that only a few patients with metastatic disease achieve cure, there have been significant improvements in palliative mCRC management. Within a short period of twenty years, the patient with metastatic colorectal cancer (mCRC) who, if left untreated had an average of 6 months overall survival, could view median overall survival reaching 30 months or even a real, however small chance of cure for very selected cases. (12, 13)

These advances in the management of mCRC occurred mainly through the emergence of different lines of chemotherapy treatment. From the isolated use of 5-fluorouracil, fluoropyrimidine, it evolved to its combination with folinic acid, enzymatic cofactor, tactic that increased survival from 9 months to 12 months with significant decreasexin toxicities.(12)

The next step was the emergence of other lines of therapy through the use of new chemotherapeutic drugs such as oxaliplatin and irinotecan. Oxaliplatin is a platinating agent that can directly damage CCR cell DNA with potentiated effect when combined with a fluoropyrimidine. Irinotecan is an inhibitor of topoisomerase I, being able to damage DNA by preventing topological stresses from being corrected and leading to fragmentation of single and double strands of DNA. The combination of these drugs with fluoropyrimidine, as in the Folfox and Folfiri regimens, and the sequential use of these regimens allowed the median overall patient survival to reach nearly two years. (12)

Another milestone in the management of mCRC was the introduction of biological agents such as monoclonal antibodies and anti-EGFR (cetuximab and panitumumab) and anti-VEGF (bevacizumab, aflibercept and regorafenib) tyrosine kinase inhibitors. (13-16) These medications can enhance the efficacy of conventional chemotherapy regimens or increase overall survival when monotherapy. The sequential use of all available weaponry has led us to imagine that mCRC could become a long-term chronic disease or in some cases even curable with associated surgery.

However, initial expectations could not be confirmed. Fewer than 10% of patients with mCRC can be cured by combined surgery and chemotherapy. (17) Anti-EGFR agents have restricted benefit to only 40% of patients whose tumors have the EGFR pathway without mutation in several enzymes, being K-ras the principal one. (18, 19) Even for patients with tumors without activating mutation along the EGFR pathway, the addition of anti-EGFR agents is not able to bring about cure or prolonged disease control. For patients with mutated K-ras tumors, there is no benefit from the use of anti-EGFR agents, and the use of cetuximab is actually deleterious in these patients. (18) Thus, the scenario is much more limited for those patients having tumors with mutation in the EGFR pathway.

There is no biomarker yet that allows choosing patients who benefit from the use of bevacizumab or aflibercept, which prevents the rational use of financial resources to afford very expensive treatment. Even regorafenib, the newest multi-kinase drug against mCRC, lacks biomarker that indicates who should be treated and who may be spared from the side effects of this medication. In fact, all these new medications have their use frankly limited by the cost. (20)

Despite these limitations, studies continue to uncover new strengths and weaknesses of mCRC. One drug that may perform anti-tumor activity is metformin. It is estimated that 100 million people use metformin daily to control type II diabetes. Early epidemiological studies have shown a reduction in the amount of cancer in individuals taking metformin compared to any other drug. (21, 22) Similarly, a recent study of breast cancer in patients with type 2 diabetes who were taking metformin showed a significant increase in pathological complete response (23) and a large phase III study to evaluate metformin as an adjuvant is under development. (24) These studies use the metformin dose commonly used in the clinic for type II diabetes control. , precluding a phase I clinical study. (25) Preliminary data from a randomized study conducted and led by our group comparing paclitaxel with or without metformin as a palliative treatment of cisplatin-refractory head and neck cancer patients has encouraging data (<http://www.saude.sp.gov.br/resources/instituto-desaude/homepage/pdfs/pdfs-em-geral/caderno_of_summary_site_ppsus.pdf> ).

The mechanism by which metformin exerts its effects is unclear; however AMPK inhibition of the mTOR pathway is one of the main mechanisms pointed out. (26) This fact may occur by the phosphorylation of TSC2. (27) And that the TSC1 complex -TSC2 negatively regulates cell growth by acting upstream of mTOR to cause this inhibition. (28) In several solid and hematological tumors, activation of the mTOR pathway plays an important role in tumor biology, conferring greater aggressiveness or refractoriness to anti-neoplastic therapies. Some blockers of this route have been tested in various oncology scenarios with consistent results. The first described inhibitor of mTOR is rapamycin.

Metformin inhibits mitochondrial respiratory chain complex I, resulting in reduced ATP production and consequently AMPK activation (29-32). Thus, activation of this signaling pathway is necessary to reduce circulating glucose levels. (30) However, activation of AMPK not only programs metabolism, but also alters proliferation and cell death suggesting that this drug may be useful in cancer treatment (33)

The insulin signaling pathway is activated when nutrients are available and the AMPK pathway is activated when the cells suffer from the lack of carbon sources. (32) In mammals, insulin promotes the synthesis of lipids, proteins and glycogen and the AMPK inhibits these biosynthetic pathways. The effect of insulin on protein synthesis is mediated in part by activation of the mTOR pathway via TSC2 phosphorylation (Figure1), and the AMPK activation causes TSC2 phosphorylation at sites different from the ones phosphorylated by Akt and inhibits mTOR. (27, 34)

There also seems to be a direct connection between these two ways. In some tissues, such as the cardiac muscle, insulin antagonizes AMPK activation (35, 36) apparently through Akt activation. (37) Apparently this occurs through phosphorylation by Akt serine residues 485 or 491 in the α1 or α2 subunits, respectively, of AMPK, which antagonizes AMPK activation through Thr 172 phosphorylation by LKB1. (38) It is not established yet, whether this mechanism also occurs in other tissues. (32)


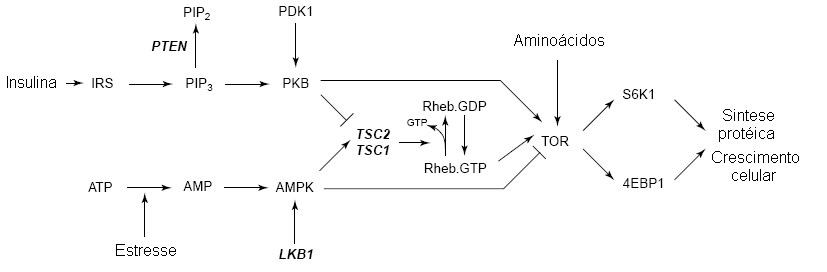


Figure 1: Interrelation between IRS / PI3 kinase / Akt (PKB) / mTOR pathways and AMPK pathway

Preclinical data from our group showed synergistic antitumor effect of irinotecan combined with an mTOR inhibitor, rapamycin, on the growth of graft colon cancer HT29 strain in immunodeficient mice (SCID mice). (39) As shown in Figure 2, mice treated with irinotecan and rapamycin at doses that reduce tumor growth "in vivo" for each agent alone. Although tumor growth was reduced in mice treated with each agent alone, tumors still increased approximately fivefold. In contrast, tumor growth in animals treated with the combined drugs was completely blocked. These data demonstrate that the combined use of rapamycin with irinotecan dramatically reduces tumor growth “in vivo”.


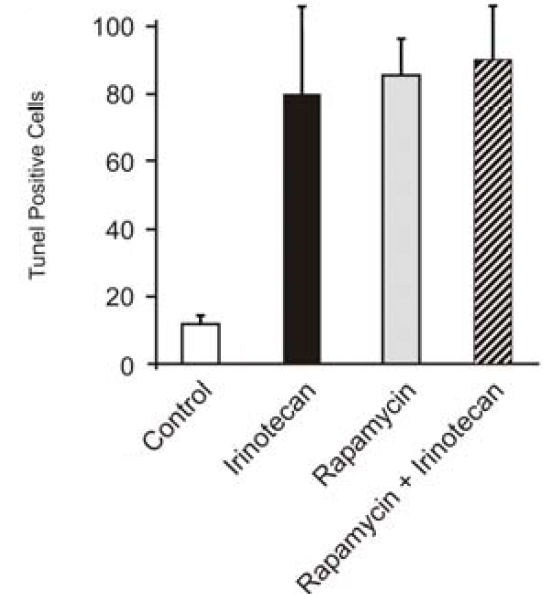


Figure 2. Association of irinotecan and mTOR (rapamycin) inhibitor in preclinical study

1.2 Metformin Overview

Metformin (DCI; marketed as Glifage, Dimefor, Glucoformin, Glucophage, among other brands, and as a generic medicine) is an oral antidiabetic of the biguanide class. The development of the biguanide class was derived from the study of the effects of the plant Galega officinalis, widely used in Europe since the Middle Ages as a popular treatment for diabetes polyuria. (40) Later the chemical compound responsible for the effect was discovered. The plant's hypoglycemic - called galegina - was a guanidine derivative. Guanidine itself is too toxic to use as a medicine, but the development of derivative agents persisted, and in 1957 the first scientific description of metformin was published. (40, 41) Metformin first entered clinical use in France in 1979; in the United States, it was approved only in late 1994, due to longstanding concerns about the safety of biguanides.

### CHEMICAL STRUCTURE


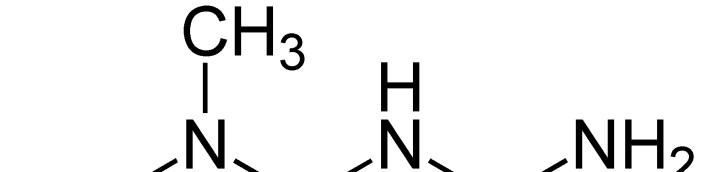

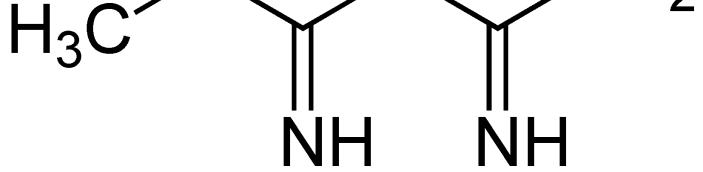


Figure 3 - Chemical structure of metformin

MECHANISM OF ACTION

  A study published in 2001 showed that metformin stimulates the function of an enzyme called AMPK, which plays an important role in lipid and glucose metabolism, a protein kinase that is capable of inactivating mTOR, a protein involved in growth regulation, proliferation and cell survival.

EXPERIMENTAL ANTI-TUMORAL ACTIVITY

  Metformin has recently demonstrated antiproliferative and proapoptotic effects in different cell lineages, including breast, prostate, and colon cancer. (43-47)

POPULATION STUDIES AND RETROSPECTIVE COHORTS

  Population studies have shown that metformin use decreases the incidence of cancer and cancer-related mortality in diabetic patients. (21, 22) It has recently been observed in a retrospective study that diabetic breast cancer patients receiving metformin and neoadjuvant treatment for breast cancer had a higher response rate than diabetic patients who did not receive metformin. (23)

ADVERSE EFFECTS

Gastrointestinal

The most common adverse effects of metformin are gastrointestinal in nature - nausea, vomiting, diarrhea, gas, cramps and lack of appetite - and are most common at the beginning of treatment or after a dose increase. Metformin seems to cause gastrointestinal discomfort more frequently than most other antidiabetic agents (48). In a US clinical study of 286 patients, more than half of those receiving metformin reported diarrhea, compared with just over 11% of those receiving placebo, and a quarter of patients reported nausea or vomiting, compared with just over 8% of those taking placebo.

Although metformin is well tolerated by most people, its gastrointestinal effects can be quite uncomfortable; it is possible to avoid them by starting treatment at a low dose and gradually increasing it. Abdominal discomfort after chronic (prolonged) use is rare.

Prolonged use of metformin is associated with an increase in blood homocysteine ​​levels (49) and malabsorption of vitamin B12 (50, 51). The higher the metformin dose and duration of use, the higher the incidence of vitamin B12 deficiency; some authors recommend prevention strategies (52).

Lactic Acidosis

The most serious potential adverse effect of metformin is lactic acidosis or lacto acidosis. However, it is quite rare, and seems to occur only in people with impaired liver or kidney function.

Another biguanide, phenformin, has been withdrawn from the market in several countries (including the United States, Portugal and Brazil) due to an unacceptable risk of causing lactic acidosis. Metformin, however, is safer, and it has been shown that it does not increase the incidence of lactic acidosis when known contraindications are observed (53).

1.3 Overview of irinotecan


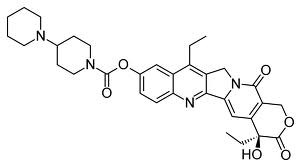
 Figure 4- Chemical structure of irinotecan

Irinotecan (CPT-11) is a semisynthetic analogue of camptothecin, originally isolated in China from an ornamental plant known as Camptotheca acuminata. It is a chemotherapeutic agent that specifically causes cell death at the S phase by binding to topoisomerase I (Topo I). The discovery and synthesis of this drug occurred in Japan in 1983, and only recently, the potent antitumor activity of irinotecan against numerous tumors has become clear. (54)

CPT-11 is a prodrug converted by carboxyl esterase into its active form SN-38, which has been used in cancer patients. Phase II studies showed an objective response rate in 32% of colorectal cancer patients. Subsequently, phase III studies consolidated the role of CPT-11 in first-line treatment in combination with fluorouracil and folic acid for the treatment of metastatic colorectal cancer (55).

MECHANISM OF ACTION

Irinotecan interacts with a topoisomerase I - DNA complex and has a specific S - phase cytotoxicity. (54) Topoisomerases reduce DNA torsion and control supercoiling that occurs in specific regions of DNA, enabling its transcription and replication. Irinotecan cleaves the phosphodiester binding of DNA, and forms a covalent binding of the enzyme to DNA, which allows another strand (double or single) of DNA to pass through the ends of the DNA. Topoisomerase I binds at this cleaved end of DNA and the complex formed Topo I-irinotecan - DNA, but alone is not lethal to cells. However, collisions with advanced replication forks cause DNA strand breaks, leading to an irreversible delay in replication and cell death. (56) The collision of the irinotecan complex with topoisomerase I with the replication process also results in delayed G2 phase by signaling the presence of DNA damage to the S-phase checkpoint mechanism of the cell cycle. (57) High concentrations of irinotecan, in non-S-phase cells, also destroy cells and this mechanism of cell death is transcriptionally mediated by DNA damage through apoptosis. (58)

RESISTANCE MECHANISMS TO IRINOTECAN

INTRACELLULAR LEVEL DECREASE OF IRINOTECAN BY INCREASED DRUG EFFLUX

Resistance to multiple drugs is the major obstacle encountered by cancer chemotherapy, it is characterized by increased expression of ATP-linked transmembrane transporters (ABC – ATP binding Cassete) such as P-glycoprotein and protein associated to multidrug resistance (MRP), which carry chemotherapeutic agents out of the cell. (59) P-glycoprotein and MRP are involved in the active efflux of SN-38 and irinotecan. (60, 61)

TOPOISOMERASE I LEVELS

Topoisomerase I is the cellular target of irinotecan, and it is acceptable that topoisomerase I cellular levels be proportional to cytotoxic effects. This idea derives from experiments with mammalian cell lines. (62, 63) Irinotecan-resistant cell lines gradually became resistant through continuous irinotecan treatments, and the total activity of topoisomerase I was reduced when compared to cell-sensitive cell lines. (64) Theoretically the expression of topoisomerase I found in various tumors may be indicative of irinotecan sensitivity. Increased levels of topoisomerase I are demonstrated in colon cancer. (64, 65) The levels of topoisomerase I found in colorectal tumors are high compared to the normal colonic mucosa suggesting a causal relationship with favorable therapy. (64)

TOPOISOMERASE MUTATIONS I

Topoisomerase I mutations are found in cells resistant to Irinotecan. (66, 67) These mutations are found in areas that may alter the DNA cleavage or DNA interactions with irinotecan.

NFΚB AND IRINOTECAN

Most chemotherapy acts by inducing the apoptotic pathway. The resistance in apoptosis induction is the main mechanism by which the cancer cell protects against cell death. (68) The activation of NFκB by TNF-α, radiation ionizing agents and chemotherapy, leads to inhibition of the apoptotic response induced by stimulation in fibrosarcoma cells. (69) It has recently been reported that the inhibitor of topoisomerase I CPT-11/SN-38 activates the pathway of NFκB in cells of fibrosarcoma and hepatocellular carcinoma. (70)

PI 3-KINASE AND IRINOTECAN

Initial studies in human hepatocellular carcinoma indicate that PI3- kinase blockade increases SN-38-induced apoptosis. (71) However, the molecular mechanisms data involved in PI3 kinase activation are still unknown.

Thus, the combination of metformin and irinotecan could prevent tumor leakage by activating mTOR.

2. Purpose and justification

In this study we are planning to evaluate the safety and efficacy of metformin combined with irinotecan in patients with recurrent or metastatic colon and rectal cancer who have failed all available conventional chemotherapy regimens and who are not candidates for surgical treatment with curative intent. These patients do not have valid therapeutic options approved by national regulatory agencies.

3. Objectives and Outcomes

Primary, secondary and exploratory objectives will be tested in the population of patients with stage IV or recurrent colorectal cancer who:

• Develop disease progression after systemic treatment including fluoropyrimidines, oxaliplatin, irinotecan, and patients without the k-ras gene mutation were also resistant to cetuximab or panitumumab.

3.1 Primary Objectives

To evaluate disease control rate (stable disease + partial response + complete response) at week 12 of treatment by RECIST v1.1 (72) and the safety of metformin + irinotecan combination in patients with stage IV colon or rectum cancer who failed chemotherapy regimens containing oxaliplatin, fluoropyrimidine, irinotecan and those with wild Kras who also failed to use cetuximab or panitumumab.

3.2 Secondary Objectives

To evaluate response duration, objective response, progression free survival (PFS), and overall survival (SG). The quality of life will be evaluated (EORTC QLQ-C30 questionnaire).

3.3 Outcomes

3.3.1. Primary outcome

The primary endpoint assessed will be disease control rate at week 12 of treatment by the RECIST v1.1 criterion in the population studied. (72)

Objective response is defined as the proportion of patients with complete response (CR) or partial response (PR) confirmed according to the RECIST v1.1 criteria, related to the total population of included patients.

3.3.2. Secondary outcomes

Secondary outcomes include overall response rate (ORR), duration of response, PFS, OS, quality of life, and tolerability. The disease control rate corresponds to the objective response rate plus the rate of stable disease patients.

Progression is defined as an increase of 20% or more in the sum of the largest target lesion diameters, or the appearance of new lesions, according to RECIST v1.1, or death from any cause.

The duration of the response will be measured from the time of CR or PR documentation (whichever is first recorded) to the date of disease progression documentation, with reference to the smallest measures recorded during the course of treatment.

The duration of stable disease will be measured from the moment therapy is started until the criteria for progression are met. The duration of stable disease is conceptually identical to the time to disease progression.

Toxicity will be classified using standard terminology criteria from the American National Cancer Institute for Adverse Events, Version 4. (NCI (73) AE v4). The performance index will be ranked according to the ECOG (US Cooperative Eastern Oncology Group) criteria.

4. Patient Selection Criteria

The target population includes adult patients with histologically confirmed (stage IV or recurrent) mCRC who have failed chemotherapy regimens containing oxaliplatin, irinotecan and fluoropyrimidine and cetuximab or panitumumab if wild Kras, and which have measurable or evaluable disease.

The investigator should ensure that patients meet all inclusion criteria and none of the exclusion criteria to enter the study. In addition, prior to the first dose of study medication, all laboratory biochemistry and hematology values must meet the inclusion criteria.

4.1 Inclusion criteria:

- Patients aged 18 years and over;

- Patients with colonic or rectal adenocarcinoma with recurrent or metastatic disease refractory to oxaliplatin, irinotecan, fluoropyrimidine-based treatment and, if wild Kras, should also be resistant to cetuximab or panitumuabe; We define refractoriness as radiological progression during or within six months of termination of treatment containing all these drugs;

- Not a candidate for any treatment with curative intent, including surgery and radiation therapy;

- Measurable or evaluable disease according to the RECIST 1.1 criteria; (72)

- ECOG 0-2;

- Appropriate haematological functions (hemoglobin ≥ 10 g / dL [5.6 mmol / L]; neutrophil count ≥ 1,500 / mm3 and platelet count ≥ 100,000 / mm3), adequate renal function (serum creatinine less than 1.5 times the limit). normal liver function) (bilirubin less than 1.5 times the normal limit; aspartate aminotransferase and alanine aminotransferase less than five times the normal limit when there is liver metastasis, or 2.5 times the normal limit when the liver is free of metastasis);

4.2 Exclusion Criteria:

- Patients with known hypersensitivity to and / or prior irinotecan and metformin therapy;

- Chronic treatment with immunosuppressive agent or corticosteroids at a dose greater than 10 mg / day of prednisone;

- Patients with known central nervous system (CNS) metastases;

- Patients with a previous diagnosis of another neoplasm, except for basal cell carcinoma of the skin and noninvasive cervical cancer;

- HIV-positive patients will be accepted if they do not have active AIDS-defining illness or AIDS-associated condition that compromises the safety of patients, with stable antiretroviral treatment and reconstituted immunity (CD4 lymphocyte count ≥200 / mm³, regardless of viral load). ;

- Chronic or acute active infection (except known HIV infection with no relevant immune repercussions);

- Patients with active bleeding or bleeding diathesis;

- Patients with any uncontrolled serious medical condition or other conditions that may affect their participation in the study;

- Patients who are taking another study medication or who have received study drugs for less than 4 weeks;

- Patients who are pregnant or nursing;

- Diabetic patients on metformin treatment;

- Absolute contraindication to loperamide use.

5. Study Design

This is a prospective phase II single arm clinical study designed to evaluate the effect of metformin with irinotecan for patients with progressing mCRC after previous treatment. The primary end point is the disease control rate (stable disease + partial response + complete response) at week 12 of treatment. The efficacy assessment will be by intention to treat. The metformin and irinotecan combination will be administered continuously until disease progression by the RECIST v1.1 criteria or in case of severe toxicity or withdrawal of consent.

This study will be conducted in Barretos Cancer Hospital. The tumor response will be evaluated in the sequence below, counting from the first day of medication infusion: 6th week, 12th week, 18th week, 24th week and every 12 weeks after, using consistent imaging techniques (CT) or MRI if patients are contraindicated for CT. The evaluation will be made by the researchers through RECIST v1.1. Discontinuation of metformin medication for three days prior to three days after infusion of any iodinated contrast media for radiological studies will be allowed at the discretion of the radiology team. This frequency of examinations is similar to that used in third-line mCRC clinical studies and arises from the need to follow more closely patients whose disease has more aggressive behavior and a greater chance of disease progression in the first few years.

Toxic effects will be analyzed according to NCI CTCAE version 4.0. (73)

The study period for each patient is defined as the day of patient inclusion until:

• The day when an event (disease progression as defined by RECIST, or death) is observed.

• The day when the patient discontinues treatment due to an unacceptable adverse event, withdrawal of consent or discontinuation of the study, or for any other reason than 21 days.

All those who discontinue treatment for any reason will be followed for survival assessment.

Patients who demonstrate disease progression during the study or who discontinue the study drug for any reason other than disease progression and withdrawal of consent (ie adverse event, administrative reasons, etc.) will be subjected to survival monitoring. The investigator or designated member will contact the patient monthly for survival information and additional antineoplastic therapies.

6. Therapeutic regimens, expected toxicity, dose modifications.

6.1 Administration of medicines

Patients will be asked to take their metformin tablets daily twice a day at approximately the same time each day and should be instructed to swallow the whole tablets with a full glass of water, not chew them. All patients should avoid eating grapefruit during the study as this may increase and prolong exposure to the study drugs.  The vomited, missed or missed doses should not be re-administered and patients should take the next dose on time following the schedule. Interruption of metformin medication for three days prior to infusion of any contrast medium for radiological studies will be allowed at the discretion of the radiology team.

Metformin will initially be administered at a dose of 1500mg / day orally for 7 days, beginning on the first day of chemotherapy infusion. Patients with good tolerance (absence of grade 3 or 4 adverse events) may have metformin dose escalated to 2500 mg / day on the eighth day of the first cycle. This metformin dose and escalation regimen is in line with the current design of metformin research on cancer treatment, either alone or in combination with conventional chemotherapies. (25) These doses are the commonly used and recommended for the treatment of patients with diabetes. type II, whether they have cancer or not.

Patients will receive irinotecan 350 mg / m^2^ intravenously, diluted in 500 mL 5% dextrose solution, in continuous infusion lasting 3 hours on D1 every 3 weeks, exactly as is done in the routine care of this service. ( 75) Prior to irinotecan infusion, the patient will receive the following medications:

1. Dexamethasone 8 mg intravenously 15 minutes before infusion;

2. Ondansetron 8 mg intravenously 15 minutes before infusion;

3. Atropine 0.25 mg subcutaneously.

6.2 Dose Adjustments

M ETFORMIN

Patients will initially receive 1500 mg / day metformin for 7 consecutive days, and on day 8 they will be clinically evaluated for toxicity. Patients without evidence of serious adverse events (grade 3 or 4) will have metformin dose escalated to 2500 mg / day. In case of intolerance, the dose will be reduced to 1500mg / day. Dose reduction to 1000mg / day will be allowed in cases of grade 3 or 4 toxicity in patients taking metformin 1500mg / day, as well as dose reduction from 2500mg / day to 1500mg / day will also be permitted in cases of intolerance. .

Recovery of acceptable toxicity levels should occur within 3 weeks to allow further study. Those with toxicity that lasts for more than 3 weeks preventing re-introduction of the study drug will be excluded. Reduction of even metformin to level -1 will be allowed (table 1).

Table 1: Available dose levels for metformin.

| Level | Daily dose | Administration |
| --- | --- | --- |
| 1 | 2500 mg / day | 1 x 1500 mg  1 x 1000 mg |
| 0 | 1500 mg / day | 1 x 1000 mg 1 x 500 mg |
| - 1 | 1000 mg / day | 1 x 500 mg  1 x 500 mg |

Doses will be reduced for haematological and other adverse events. Dose adjustments are made according to the system that shows the highest degree of toxicity. Adverse events will be classified using the National Cancer Institute Common Terminology Criteria for Adverse Events Version 4 (NCI CTCAE). (73)

Table 2: Dose modifications for metformin-associated toxicities

| Toxicity | Grade 1 | Grade 2 | Grade 3 | Grade 4 |
| --- | --- | --- | --- | --- |
| Non hematologic | Keep the same  dose level | Maintain the same dose level. The dose may be discontinued until toxicity is ≤ 1 or has returned to baseline at the investigator's discretion. Then resume treatment at the same dose level. | Drop the dose until the toxicity is grade ≤ 1 or has returned to baseline, then resume treatment at the same dose level or reduce the dose to level 1 at the investigator's discretion **. | Drop the dose until the toxicity is grade ≤ 1 or has returned to baseline, then reduce the dose to level -  1 or discontinue treatment at the discretion of the investigator ^#^. |
| Hematologic | Keep the same  dose level | Maintain the same dose level *. | Discontinue the dose until the toxicity is ≤ 1 or has returned to baseline, then resume treatment at the same dose level ***. | Drop the dose until the toxicity is grade ≤ 1 or has returned to baseline, then reduce the dose to level 1 and resume treatment ^##^. |

* To start another cycle, the neutrophil count must be greater than 1,500 / mm ^3^, platelet count greater than 100,000 / mm ^3^and hemoglobin greater than or equal to 9 g / dL. ** Patients with grade 3 hypoglycemia, fatigue / asthenia who require more than 2 weeks without metformin administration to return to grade ≤ 1 toxicity should have their dose reduced to level -1.

*** Grade 3 lymphopenia and leukopenia do not require the dose to be suspended or reduced. Grade 3 recurrent neutropenia or thrombocytopenia that persists for at least 7 days requires dose reductions to level -1 in the next cycle.

Grade 4 haemorrhage requires discontinuation of study drug.

Grade 4 lymphopenia without other dose limiting events (opportunistic infection) does not require dose reduction.

If treatment discontinuation is required and the patient is unable to resume treatment within ≤ 28 days, treatment should be discontinued and the patient will continue to be followed for tumor response rate assessment and survival follow-up.

IRINOTECAN

Dose reduction in the patient will be allowed depending on the type and severity of the toxicity found. The dose levels of irinotecan are 125 mg / m², 100 mg / m² and 75 mg / m². The dose-limiting toxicities of irinotecan are related to neutropenia, alopecia, nausea, severe diarrhea, hypersensitivity reactions, asthenia and myalgia. Considering that the clinical toxicity most commonly related to irinotecan intolerance is diarrhea, the patient will be instructed to start treatment with loperamide when experiencing grade 1 diarrhea in order to avoid severe diarrhea and its complications.

| Toxicicity | Grade 1 | Grade 2 | Grade 3 | Grade 4 |
| --- | --- | --- | --- | --- |
| Non haematological | Keep the same dose level | Reduce dose in 25 mg/m²and on the next cycle keep the same dose level | Hold the dose until the toxicity is ≤ 2, then resume treatment with a reduced dose of 25 mg / m² (minimum dose of 75 mg / m2) | Hold the dose until the toxicity is ≤ 1 or has returned to baseline, then reduce the dose by 50 mg / m² (minimum dose 75 mg / m2)  or discontinue treatment, at the discretion of the investigator |
| Haematological | Keep the same dose level | Reduce dose in 25 mg/m² and on the next cycle keep the same dose level | ² Hold the dose until the toxicity is ≤ 2, then resume treatment with a reduced dose of 25 mg / m² (minimum dose of 75 mg / m2) | Hold the dose until the toxicity is ≤ 1 or has returned to baseline, then reduce the dose by 50 mg / m² (minimum dose 75 mg / m2)  or discontinue treatment, at the discretion of the investigator |

 DOSE SCALE

Every effort should be made to reschedule the metformin dose to the starting dose level. This applies to any of the dose reductions due to haematological or non-haematological toxicities. The dose should be rescheduled if the following criteria are met at least one month after dose reduction:

• All non-haematological toxicities Grade ≥ 2 reach Grade ≤ 1

• All Grade ≥ 3 hematologic toxicities reach Grade ≤ 1

• Or, alternatively, all haematologic toxicities and non-haematologica Grade ≥ 3 are resolved to a Grade ≤ 2 or can be controlled with supportive therapy.

6.3 Duration of Therapy

Treatment cycles will be repeated until progression or unacceptable toxicity occurs as previously described. Patients may also discontinue the drug in the following instances:

• Concomitant disease, which could, in the opinion of the investigator, affect patient safety, ability to comply with treatment, or primary study outcomes;

• Request by the patient through withdrawal of consent;

• The patient missed or did not follow up;

• The need for other antineoplastic therapy not specified in the protocol.

• If there are more than two response assessments, irinotecan therapy may be discontinued at the investigator's discretion.

6.4 Concomitant Therapy

No other treatment under approved antineoplastic research will be allowed during the study period, including chemotherapy, biological response modifiers, hormone therapy, immunotherapy, surgery or radiotherapy (except for single-dose radiation as a pain reliever). No other investigational drugs may be used during treatment in this protocol, and concomitant participation in another study is not allowed. If the patient requires radiation therapy while in the study, metformin may be discontinued one day before and resumed the day after radiation therapy.

Supportive care may include antiemetics to limit treatment-related nausea and vomiting, as well as loperamide for diarrhea. Anti-inflammatory drugs or narcotic analgesics may be offered as needed. Red cell concentrate or platelet transfusions should be administered as clinically indicated. The use of colony stimulating factors is allowed only in the case of neutropenic fever.

7. Schedule visits and evaluations

7.1 Before starting treatment

The following screening investigations are required two weeks prior to study initiation to assess patient eligibility:

• Signing of the Informed Consent Form

• Medical history, including diagnosis and treatment of mCRC (dose and duration), history of other diseases (active and resolved), concomitant diseases and medications, analgesic and demographic use.

• Complete physical examination, ECOG performance index (PS), body weight and height (screening only), and vital signs (ie temperature, blood pressure, heart rate, and respiratory rate).

• Pregnancy test: serum pregnancy test for all women of childbearing age and not in menopause.

• Hematological and blood biochemistry as described in section 7.4.

• Quality of life assessment using the EORTC QLQC30 questionnaire translated into Portuguese.

The following radiological examinations are required up to three weeks before starting the study drug:

• Abdominal computed tomography (CT) or nuclear magnetic resonance

(MRI) of the abdomen and chest tomography

• Other tests may be performed as directed by individual patient.

• Tumor response will be assessed at 6 weeks, 12 weeks, 18 weeks after initiation of therapy, and thereafter every 12 weeks until disease progression. The evaluation criteria performed by the researchers will follow the concepts proposed by RECIST v1.1.

7.2 During treatment

The following investigations are required during treatment:

• Medical assessment (including medical history, concomitant medications, analgesic use, adverse event assessment, physical examination, ECOG PS determination, vital signs, and body weight) on days 1 and 8 of cycle 1 and the first day of subsequent cycles.

• Biochemical, hematological analysis on days 1 and 8 of the first cycle and on the first day of subsequent cycles. If the patient has haematological, renal or hepatic toxicity ≥ grade 3, then laboratory tests should be repeated weekly until reduction to ≤ grade 1.

• Chest CT, total abdomen CT and chest X-ray will be performed during patient screening. If the patient has contraindications to the use of tomographic contrast, non-contrast-enhanced chest tomography and total abdomen magnetic resonance imaging will be performed. For response evaluation, tomography of the tumor sites will be performed every 6 weeks until the 18th week of the study, and then every 12 weeks. The same method and technique should be used to characterize each lesion identified and reported on baseline examination and during the study treatment period. Imaging-based evaluation, in addition to clinical examinations , are the techniques required when both can be used to evaluate the antitumor effect of treatment.

• Quality of life assessment with the EORTC QLQ-C30 and CR29 questionnaires translated into Portuguese will be performed on screening, on the first day of cycles 3, 6, 9, every four cycles onwards.

7.3 After the end of treatment (follow up)

• Medical (including medical history, concomitant medications, analgesic use, physical exams, ECOG PS determination, vital signs and body weight) and laboratory evaluation will be performed 28 days after protocol exclusion. Patients withdrawn from the study will be followed for survival assessment.

7.4 Study Summary

Table 3 Visit Evaluation Schedule

| Required Investigations | Tracking | Day  1  Cycle  1 | Day  8th  Cycle  1 | Day  1  Cycle  2 | Day  1  Cycle  3 | Day  1  Cycle  4 | Day  1  Cycle  5th | Day  1  Cycle 6 | Subsequent cycles | End of treatment |
| --- | --- | --- | --- | --- | --- | --- | --- | --- | --- | --- |
| Medical evaluation |  |  |  |  |  |  |  |  |  |  |
| Clinical symptoms | X | X | X | X | X | X | X | X | X | X |
| Physical exam | X | X | X | X | X | X | X | X | X | X |
| Vital signs, body weight and PS | X | X | X | X | X | X | X | X | X | X |
| Concomitant Drugs | X | X | X | X | X | X | X | X | X | X |
| Adverse Event Assessment | X | X | X | X | X | X | X | X | X | X |
| Hematology |  |  |  |  |  |  |  |  |  |  |
| Blood count  Complete | X | X | X | X | X | X | X | X | X | X |
| Biochemistry |  |  |  |  |  |  |  |  |  |  |
| Creatinine / Urea / Glucose | X | X | X | X | X | X | X | X | X | X |
| AST / ALT / bilirubin / alkaline phosphatase / cholesterol and triglyceride levels | X | X | X | X | X | X | X | X | X | X |
| Insulin, CEA, whole blood | X | X | X | X | X | X | X | X | X | X |
| Sodium, Potassium, Magnesium, Calcium,  phosphor | X | X | X | X | X | X | X | X | X | X |
| Radiology ^*^ |  |  |  |  |  |  |  |  |  |  |
| Chest / abdomen CT | X |  |  |  | X |  | X |  |  |  |
| Quality of life assessment | X |  |  |  | X |  |  | X | X | X |
| Pregnancy test | X |  |  |  |  |  |  |  |  |  |

Chest CT, total abdomen CT will be performed during patient screening. For evaluation of response will be performed tomography of the tumor sites and chest X-ray at 6 weeks, 12 weeks and every 12 weeks onwards.

8. Evaluation Criteria

8.1 Response Rate

All eligible patients will be included in the response rate calculation. The subgroups that designated a response category (CR, PR, ED, and disease progression - PD; see definitions below) are all patients who received at least one dose of study medications.

Radiological response

Response and progression will be assessed in this study using the new international criteria proposed by the RECIST v1.1 committee (Response Evaluation Criteria for solid tumors). Changes only in the larger diameter (one-dimensional measurement) of tumor lesions are used in the RECIST v1.1 criteria (Appendix

2).

9. Toxicity Assessment

Adverse Events (EA) will be classified using the Common Terminology Criteria of the American Cancer Institute for Adverse Events Version 4.0 (NCI - CTC AE) (73). All patients will be evaluated for study drug toxicity from the time of their first treatment dose until at least 28 days after the last dose.

10. Statistical Considerations

10.1 Statistical design and sample size

This is a prospective, unicenter, single-stage, phase 2, two-stage study to investigate the efficacy and safety of the combination of irinotecan and metformin in patients with metastatic colorectal cancer, to be a promising regimen for further extension to a phase 3 trial. The primary end point is the disease control rate at week 12 of treatment.

Simon's optimized two-stage procedure will be used for the sample calculation. (76) Considering a DCR of 13,2% on week 12 with placebo in third line, we believe that it would be clinically significant to increase this DCR to 26,7% Considering an alpha error of 10% and a beta error of 20%, we planned to enroll 41 patients. This study would be considered positive if at least 9 patients had disease control on week 12 (16). We believe this increase in the rate of disease control is feasible in light of the preliminary analysis of preclinical data presented by our group and other ongoing clinical protocol in our services that assesses metformin combined with chemotherapy (PPSUS 2011 Project - FAPESP Support - http://clinicaltrials.gov/show/NCT01333852 ).

Secondary objectives will be safety and tolerability, quality of life, SLP, and SG. Whole blood and fractions will be collected and deposited in our biobank to enable future evaluation of host and tumor serum response and prognostic markers. (77) Efficacy outcomes will be analyzed according to intention-to-treat (ITT).Safety outcomes will be assessed across the population exposed to study medications.

11. Recruitment and Study Duration

The estimated recruitment for this study is 41 patients. The study recruitment period will be estimated at 22 months, and all patients will remain on treatment and follow-up until disease progression. The total period of the study will last two years and may be extended as the recruitment rate is lower than expected, after discussion with internal monitoring bodies, Barretos Research Ethics Committee.

There will be continuous monitoring of the results and progress of the study by the Research Ethics Committee, as routine, in order to ensure the safety of the research subjects. If necessary, the study may be discontinued early, either because of futility, insecurity or early attainment of its positive outcomes at the end of the first phase of the study. Those patients who progress or are excluded from the study will be monitored for survival assessment.

The study period for each patient is defined as the day of patient inclusion until:

• The day when an event (disease progression as defined by RECIST, or death) is observed in this patient;

• The day when the patient discontinues treatment due to an unacceptable adverse event, withdrawal of consent or discontinuation of the study, or for any other reason than 21 days.

All those who discontinue treatment for any reason will be followed for survival assessment.

Patients who demonstrate disease progression during the study or who discontinue the study drug for any reason other than disease progression and withdrawal of consent (ie adverse event, administrative reasons, etc.) will have their survival accompanied. The designated investigator will contact the patient monthly for survival information and additional antineoplastic therapies.

12. Statistical Analysis

The primary end point is disease control rate at week 12, which is the sum of complete response, partial response, and stable disease in this assessment. Dichotomous efficacy outcomes, as well as patient baseline characteristics and adverse events, will be analyzed by Fisher's exact test and the Χ² test. For survival analyzes, progression is defined as radiological progression (RECIST 1.1) (72) or death from any cause. Progression free survival (SLP) and overall survival (SG) will be estimated by the Kaplan-Meier method. Other results will be reported using descriptive statistics. SPSS software version 14.0 or later as available (SPSS Inc., Chicago-IL, United States) will be used for statistical analysis of the results.

13.  Procedures and Data Collection

Data will be collected in the case report form ([CRF] supplement 1) and recorded in a specific study database at the Research Support Center of the Barretos Cancer Hospital. The original completed CRF will be sent to the principal investigator.

13.1 Clinical Records (CRF)

A CRF is required and must be completed for each included patient and sent to the principal investigator within 90 days of the patient's discontinuation .

It is the responsibility of the investigator to ensure completion, review and approval of all CRFs. The CRF must be signed by the investigator or an authorized team member (co-investigator). These signatures serve to certify that the information contained in the CRF is true. At all times, the investigator has ultimate personal responsibility for the accuracy and authenticity of all clinical and laboratory data recorded with the CRF. Patient source documents (medical records) are the patient's medical records kept at the study institution.

14. Adverse Event

14.1 Definitions

An Adverse Event (AE) is defined as any unfavorable medical occurrence or experience, in a patient or patient under clinical investigation, that occurs after administration of study medication, regardless of dose or causal relationship. This may include any unfavorable and involuntary signs (such as rash or liver enlargement), or symptoms (such as nausea or chest pain), an abnormal laboratory finding (including blood tests, X-rays or CT scans) or a disease temporarily associated with use. of protocol treatment.

An Adverse Drug Reaction (ADR) is defined as any response to the medicinal product, which is harmful and / or unexpected, related to any dose.

Response to the medicinal product (used in the definition above) means that a causal relationship between the medicinal product and the adverse event is at least a reasonable possibility, ie the relationship cannot be ruled out.

An Unexpected Adverse Drug Reaction is any adverse reaction for which the nature or severity is not consistent with the information applicable to the product (eg, Investigator Brochure).

A Serious Adverse Event (EAS) is defined as any unwanted experience that occurs with a patient, whether or not considered related to protocol treatment. A Serious Adverse Event (EAS) that is considered related to protocol treatment is defined as a Serious Adverse Drug Reaction (RASM).

Adverse events and adverse drug reactions that are considered serious are those that result in:

• death;

• Risk of death (ie the patient had an immediate risk of death at the time the reaction was observed);

• Hospitalization for more than 24 hours or prolongation of hospitalization;

• Persistent or significant disability / disability;

• Congenital anomaly / birth defect;

• Any other clinically important condition (ie, major adverse reactions that are not life threatening or result in death or hospitalization but may endanger the patient or may require intervention to prevent one of the other consequences listed above).

The progression of malignancy under the study should not be reported as an adverse event as well as death due to disease progression.

14.2 Reporting Procedure

All observed or spontaneous adverse events, regardless of the suspected causal relationship with the investigational product, will be reported as described below.

For all adverse events, the investigator should seek and obtain appropriate information both to determine the outcome of the adverse event and to assess whether he meets the criteria for classification as a serious adverse event requiring immediate notification to the Research Ethics Committee. For all adverse events, sufficient information should be obtained by the investigator to determine the causality of the study drug. Event tracking is required until the event, or its consequences, is resolved or stabilizes at an acceptable level.

Adverse events (serious or non-serious) should be recorded in CRF from the moment the patient has received at least one dose of study treatment until the patient's last visit. Serious adverse events developed within 28 days of the last administration of the investigational product should be reported immediately to the Research Ethics Committee.

In accordance with Brazilian regulatory laws, the investigator must inform the local Research Ethics Committee about additional and study-specific problems every 6 months.

15. Quality Assurance

15.1 Data Compliance Control

During the conduct of the study, periodic monitoring visits will be conducted to ensure that the GCP (Good Clinical Practice) protocol and guidelines are followed. Monitors may review source documents and imaging studies to confirm that data recorded on the CRF (Clinical Record) is accurate. The investigator and the institution will allow the source documents to be monitored and appropriate regulatory authorities to have direct access to them in order to carry out verifications. The study institution may be subject to review by the Ethics Committee.

16. Ethical Considerations

This protocol and its consent form have already been approved by the research ethics committee of the proposing institution, Barretos Cancer Hospital. The free and informed consent form was prepared in compliance with ICH GCP guidelines, local regulatory regulations and legal requirements. It is the responsibility of the investigator to obtain future approval of protocol amendments and other documents relevant to the CEP.

The study will be conducted in accordance with the International Harmonization Conference (ICH GCP) Protocol, Good Clinical Practice guidelines, and applicable local regulatory laws and requirements (RDC 39-2008 ANVISA; RDC 196-1996; RDC 466-2012) .

The investigator should ensure that each study patient, or legal representative, is fully informed about the nature and objectives of the study and possible risks associated with participation. The investigator will obtain written consent from each patient before any specific study activity is performed. The investigator will retain a copy of each consent form signed by the patient.

Considering that human biological material is expected to be stored in our Hospital (biobank), in addition to meeting the requirements of Resolution CNS No. 466/12 and complementary, we clarify that:

There will be consent of the research subject, authorizing the collection, deposit, storage and use of human biological material (present in the IC);

This material will be under the supervision of the HCB Biological Material Committee, following all the precepts already in force of this body;

We declare that any future research to be conducted with the stored material will be submitted for approval by the institutional Research Ethics Committee and, where appropriate, the National Research Ethics Commission.

16.1 Patient Protection

The responsible investigator will ensure that this study is conducted in accordance with the Declaration of Helsinki (amendments from Tokyo, Venice, Hong Kong, Somerset West and Edinburgh) or with the laws and regulations of the country providing the highest patient protection.

The protocol has been written and the study will be conducted in accordance with the Tripartite Guidelines of the International Conference on Harmonization for Good Clinical Practice (http://www.ifpma.org/pdfifpma/e6.pdf).

The protocol has already been approved by the HCB Research Ethics Committee, which considered it unnecessary to submit for evaluation by the National Research Ethics Committee (CONEP). There will be continuous monitoring of safety and efficacy results by the Research Ethics Committee (CEP-HCB), as routine, in order to ensure the safety of the research subjects. If necessary, the study may be discontinued early either due to futility, lack of security or early achievement of its positive outcomes.

16.2 Patient Identification

Patients will be identified in the protocol by their initials from Barretos Cancer Hospital. Once included in the study, they will gain additional unique identification for each study subject. All patient's personal information will be confidential.

16.3 Informed Consent

All patients will be informed of the study's intentions, possible adverse events, procedures and possible risks to which they will be exposed. They will be informed of the strict confidentiality of their data and that their medical records will only be accessible to members assigned to conduct and monitor the study, such as researchers and members of the Barretos Research Ethics Committee.

It will be emphasized that participation is voluntary and that the patient has the right to refuse further participation in the protocol at any time. This will not impair subsequent patient care. Documented Informed Consent (Supplement 2) must be obtained from all patients included in this study before they are enrolled in the study. This will be done in accordance with national and international regulatory requirements.

17. Administrative Responsibilities

It is the responsibility to store the records and medical records related to the protocol. The responsible team will comply with current legislation and international standards. These forms will be in physical form (case report form [CRF]) and will be kept under study centers for a minimum of five years.

18. Schedule

The total study time is 22 months and the table below describes the steps to be taken. The date D0 implies the day of submission to our zip code. We believe it is possible to submit in July 2013. We also believe it is feasible to include 2 patients per month. The number of eligible patients is higher, however, we seek to be conservative in order not to overload the outpatient routine of the oncology, radiology or NAP department. It is important to remember that there is a possibility that the study may be interrupted by futility until June 28, 2014, or earlier, if this decision is taken jointly between the researcher and the HCB bodies involved (CEP and NAP).

Table 4: Proposed Study schedule.

| Phase | Duration | Start* | Finish * |
| --- | --- | --- | --- |
| Inclusion and treatment of  17 patients in the 1st stage | 240 days | 01.10.2013 | May 31, 2014 |
| Time to first stage patient evaluation | 30 days | 06/01/2014 | June 30, 2014 |
| Inclusion and treatment of  24 patients of the 2nd stage | 300 days | 01.07.2014 | May 31, 2015 |
| Tumor assessment of 2nd stage patients | 30 days | June 1, 2015 | June 30, 2015 |
| Statistical analysis | 30 days | 01.07.2015 | Jul 31, 2015 |
| Article Writing | 30 days | Aug 01, 2015 | Aug 31, 2015 |

*: Estimated start and end.

19. Team and Structure

At Barretos Cancer Hospital, the multidisciplinary team is composed of members of the Clinical Research Unit (UPC-NAP) of Barretos Cancer Hospital. This unit has over six years of experience in conducting clinical studies, with over 400 patients treated in pharmacological intervention studies in oncology. As a result of UPC's work, we are the author of recent multicenter studies in clinical oncology. (25, 78-80)

Another recognition of the quality of research patient care is the election of our research center as one of five centers outside the Europe-United States axis that will receive phase I studies from the Novartis oncology translational medicine program. In addition, the Barretos Clinical Research Unit is considered a preferred partner by some pharmaceutical industries (Bristol-Myers Squib and Novartis) due to its ability to meet recruitment goals and data quality by providing study protocol patient care.

At the Barretos Cancer Hospital study center, the study will feature clinical oncology medical staff, a division of gastrointestinal neoplasms, comprised of members with extensive experience in clinical research and exclusive dedication; chemotherapy pharmacy team, responsible for handling and dispensing oral medications; radiology medical team, responsible for the evaluation of the imaging exams requested during the study; UPC-NAP team, responsible for the study and logistics coordination.

The physical space used will be the area dedicated to clinical research. This area comprises a total of 1,200 m ^2^with reception, doctor's office, negative flow chapel handling pharmacy, infusion room with 15 armchairs exclusively for research patients. This area also houses all administrative and clinical research support staff, such as coordinators and data entry.

Table 5: Team involved

| Team | Number of  participants | Hours per week |
| --- | --- | --- |
| Clinical oncologist | 08 | 80h |
| Research Nurse | 02 | 40h |
| Research Pharmacist | 02 | 40h |
| Nursing technician | 02 | 40h |
| Study Coordinator | 01 | 40h |
| Coordination Assistant | 01 | 40h |
| Data collector and digitizer | 01 | 40h |
| Statistical | 01 | 4am |
| Study Coordinator | 01 | 8pm |

20. Funding of the study

This study will be funding by Barretos Cancer Hospital.

21. References

1. Jemal, A., Bray, F., Center, MM, Ferlay, J., Ward, E., and Forman, D. 2012. Global cancer statistics. CA Cancer J Clin 61: 69-90.

2. Hanahan, D., and Weinberg, RA 2000. The hallmarks of cancer. Cell 100: 5770.

3. Zauber, AG, Winawer, SJ, O'Brien, MJ, Lansdorp-Vogelaar, I., Van Ballegooijen, M., Hankey, BF, Shi, W., Bond, JH, Schapiro, M., Panish, JF , et al. Colonoscopic polypectomy and long-term prevention of colorectal cancer deaths. N Engl J Med 366: 687-696.

4. Rothwell, PM, Wilson, M., Price, JF, Belch, JF, Meade, TW, and Mehta, Z. Effect of daily aspirin on risk of cancer metastasis: a study of incident cancers during randomized controlled trials. Lancet 379: 1591-1601.

5. Schoen, RE, Pinsky, PF, Weissfeld, JL, Yokochi, LA, Church, T., Laiyemo, AO, Bresalier, R., Andriole, GL, Buys, SS, Crawford, ED, et al. Colorectal cancer incidence and mortality with screening flexible sigmoidoscopy. N Engl J Med 366: 2345-2357.

6. Schwitalla, S., Fingerle, AA, Cammareri, P., Nebelsiek, T., Goktuna, SI, Ziegler, PK, Canli, O., Heijmans, J., Huels, DJ, Moreaux, G., et al. . Intestinal tumorigenesis initiated by dedifferentiation and acquisition of stem-cell-like properties. Cell 152: 25-38.

7. Kapiteijn, E., Marijnen, CA, Nagtegaal, ID, Putter, H., Steup, WH, Wiggers, T., Rutten, H., Pahlman, L., Glimelius, B., van Krieken, JH, et al. . 2001

Preoperative radiotherapy combined with total mesorectal excision for resectable rectal cancer. N Engl J Med 345: 638-646.

8. Sauer, R., Becker, H., Hohenberger, W., Rodel, C., Wittekind, C., Fietkau, R., Martus, P., Tschmelitsch, J., Hager, E., Hess, CF. , et al. 2004. Preoperative versus postoperative chemoradiotherapy for rectal cancer. N Engl J Med 351: 1731-1740.

9. Bosset, JF, Collette, L., Calais, G., Mineur, L., Maingon, P., Radosevic-Jelic, L., Daban, A., Bardet, E., Beny, A., and Ollier. , JC 2006. Chemotherapy with preoperative radiotherapy in rectal cancer. N Engl J Med 355: 1114-1123.

10. Sanoff, HK, Carpenter, WR, Martin, CF, Sargent, DJ, Meyerhardt, JA, Sturmer, T., Fine, JP, Weeks, J., Niland, J., Kahn, KL, et al. Comparative effectiveness of oxaliplatin vs non-oxaliplatin-containing adjuvant chemotherapy for stage III colon cancer. J Natl Cancer Inst 104: 211-227.

11. Petersen, SH, Harling, H., Kirkeby, LT, Wille-Jorgensen, P., and Mocellin, S.

Postoperative adjuvant chemotherapy in rectal cancer operated for cure. Cochrane Database Syst Rev 3: CD004078.

12. Golfinopoulos, V., Salanti, G., Pavlidis, N., and Ioannidis, JP 2007. Survival and disease-progression benefits with treatment regimens for advanced colorectal cancer: a meta-analysis. Lancet Oncol 8: 898-911.

13. Macedo, LT, Costa Lima, AB, and Sasse, AD Addition of bevacizumab to first-line chemotherapy in advanced colorectal cancer: a systematic review and meta-analysis, with emphasis on chemotherapy subgroups. BMC Cancer 12:89.

14. Vale, CL, Tierney, JF, Fisher, D., Adams, RA, Kaplan, R., Maughan, TS, Parmar, MK, and Meade, AM Does anti-EGFR therapy improve outcome in advanced colorectal cancer? A systematic review and meta-analysis. Cancer Treat Rev 38: 618-625.

15. Van Cutsem, E., Tabernero, J., Lakomy, R., Prenen, H., Prausova, J., Macarulla, T., Ruff, P., van Hazel, GA, Moiseyenko, V., Ferry, D., et al. Addition of aflibercept to fluorouracil, leucovorin, and irinotecan improves survival in a phase III randomized trial in patients with metastatic colorectal cancer previously treated with an oxaliplatin-based regimen. J Clin Oncol 30: 34993506.

16. Grothey, A., Van Cutsem, E., Sobrero, A., Siena, S., Falcone, A., Ychou, M., Humblet, Y., Bouche, O., Mineur, L., Barone, C., et al. 2013. Regorafenib monotherapy for previously treated metastatic colorectal cancer (CORRECT): an international, multicentre, randomized, placebo-controlled, phase 3 trial. Lancet 381: 303-312.

17. Masi, G., Vasile, E., Loupakis, F., Cupini, S., Fornaro, L., Baldi, G., Salvatore, L., Cremolini, C., Stasi, I., Brunetti, I ., et al. Randomized trial of two induction chemotherapy regimens in metastatic colorectal cancer: an updated analysis. J Natl Cancer Inst 103: 21-30.

18. Tsoukalas, N., Tzovaras, AA, Tolia, M., Kostakis, ID, Papakostidi, A., Pistamaltzian, N., and Ardavanis, A. Meta-analysis of predictive value of KRAS mutations in treatment response using cetuximab in colorectal cancer. J BUON 17: 73-78.

19. Jiang, Z., Li, C., Li, F., and Wang, X. EGFR Gene Copy Number as a

Prognostic Marker in Colorectal Cancer Patients Treated with Cetuximab or

Panitumumab: A Systematic Review and Meta Analysis. PLoS One 8: e56205.

20. Schrag, D. 2004. The price tag on progress - chemotherapy for colorectal cancer. N Engl J Med 351: 317-319.

21. Evans, JM, Donnelly, LA, Emslie-Smith, AM, Alessi, DR, and Morris, AD 2005. Metformin and reduced risk of cancer in diabetic patients. BMJ 330: 1304-1305.

22. Bowker, SL, Majumdar, SR, Veugelers, P., and Johnson, JA 2006. Increased cancer-related mortality for patients with type 2 diabetes who use sulfonylureas or insulin: Response to Farooki and Schneider. Diabetes Care 29: 1990-1991.

23. Jiralerspong, S., Palla, SL, Giordano, SH, Meric-Bernstam, F., Liedtke, C., Barnett, CM, Hsu, L., Hung, MC, Hortobagyi, GN, and Gonzalez-Angulo, AM 2009. Metformin and pathologic complete responses to neoadjuvant chemotherapy in diabetic patients with breast cancer. J Clin Oncol 27: 32973302.

24. Goodwin, PJ, Ligibel, JA, and Stambolic, V. 2009. Metformin in breast cancer: time for action. J Clin Oncol 27: 3271-3273.

25. Goodwin, PJ, Stambolic, V., Lemieux, J., Chen, BE, Parulekar, WR, Gelmon, KA, Hershman, DL, Hobday, TJ, Ligibel, JA, Mayer, IA, et al. 2011. Evaluation of metformin in early breast cancer: a modification of the traditional paradigm for clinical testing of anti-cancer agents. Breast Cancer Res Treat 126: 215-220.

26. Rock, GZ, Dias, MM, Ropelle, ER, Osorio-Costa, F., Rossato, FA, Vercesi, AE, Saad, MJ, and Carvalheira, JB 2011. Metformin amplifies chemotherapy-induced AMPK activation and antitumor growth. Clin Cancer Res 17: 3993-4005.

27. Inoki, K., Zhu, T., and Guan, KL 2003. TSC2 mediates cellular energy response to control cell growth and survival. Cell 115: 577-590.

28. Gao, X., Zhang, Y., Arrazola, P., Anthem, O., Kobayashi, T., Yeung, RS, Ru, B., and Pan, D. 2002. Tsc tumor suppressor proteins antagonize amino acids. acid-TOR signalling. Nat Cell Biol 4: 699-704.

29. Hundal, RS, Krssak, M., Dufour, S., Laurent, D., Lebon, V., Chandramouli, V., Inzucchi, SE, Schumann, WC, Petersen, KF, Landau, BR, et al. 2000

Mechanism by which metformin decreases glucose production in type 2 diabetes. Diabetes 49: 2063-2069.

30. Shaw, RJ, Lamia, KA, Vasquez, D., Koo, SH, Bardeesy, N., Depinho, RA, Montminy, M., and Cantley, LC 2005. The kinase LKB1 mediates glucose homeostasis in liver and therapeutic effects. of metformin. Science 310: 16421646.

31. Shackelford, DB, and Shaw, RJ 2009. The LKB1-AMPK pathway:

metabolism and growth control in tumor suppression. Nat Rev Cancer 9: 563575.

32. Towler, MC, and Hardie, DG 2007. AMP-activated protein kinase in metabolic control and insulin signaling. Circ Res 100: 328-341.

33. Shackelford, DB, and Shaw, RJ 2009. The LKB1-AMPK pathway:

metabolism and growth control in tumor suppression. Nature Reviews Cancer 9: 563-575.

34. Inoki, K., Li, Y., Zhu, T., Wu, J., and Guan, KL 2002. TSC2 is phosphorylated and inhibited by Akt and suppressing mTOR signaling. Nat Cell Biol 4: 648-657. 35. Beauloye, C., Marsin, AS, Bertrand, L., Krause, U., Hardie, DG, Vanoverschelde, JL, and Hue, L. 2001. Insulin AMP-activated antagonists

protein kinase activation by ischemia or anoxia in rat hearts, without affecting total adenine nucleotides. FEBS Lett 505: 348-352.

36. Gamble, J., and Lopaschuk, GD 1997. Insulin inhibition of 5 'adenosine monophosphate-activated protein kinase in the heart results in activation of acetyl coenzyme A carboxylase and inhibition of fatty acid oxidation. Metabolism 46: 1270-1274.

37. Kovacic, S., Soltys, CL, Barr, AJ, Shiojima, I., Walsh, K., and Dyck, JR 2003. Akt activity negatively regulates phosphorylation of AMP-activated protein kinase in the heart. J Biol Chem 278: 39422-39427.

38. Horman, S., Vertommen, D., Heath, R., Neumann, D., Mouton, V., Woods, A., Schlattner, U., Wallimann, T., Carling, D., Hue, L. ., et al. 2006. Insulin antagonists ischemia-induced Thr172 phosphorylation of AMP-activated protein kinase alpha-subunits in heart via hierarchical phosphorylation of Ser485 / 491. J Biol Chem 281: 5335-5340.

39. de Souza, KK 2007. Irinotecan activates the PI3 kinase / AKT / mTOR pathway in colon / Kellen Ketty de Souza adenocarcinoma lineage. . In Graduate Program in Medical Clinic. Campinas: State University of Campinas. 100

40. Witters, LA 2001. The Blooming of the French Lilac. J Clin Invest 108: 11051107.

41. Ungar, G., Freedman, L., and Shapiro, SL 1957. Pharmacological studies of a new oral hypoglycemic drug. Proc Soc Exp Biol Med 95: 190-192.

42. Zhou, G., Myers, R., Li, Y., Chen, Y., Shen, X., Fenyk-Melody, J., Wu, M., Womb, J., Doebber, T., Fujii. , N., et al. 2001. Role of AMP-activated protein kinase in mechanism of metformin action. J Clin Invest 108: 1167-1174.

43. Anisimov, VN, Berstein, LM, Egormin, PA, Piskunova, TS, Popovich, IG,

Zabezhinski, MA, Kovalenko, IG, Poroshina, TE, Semenchenko, AV, Provinciali, M., et al. 2005. Effect of metformin on life span and development of spontaneous mammary tumors in HER-2 / neu transgenic mice. Exp Gerontol 40: 685-693.

44. Zakikhani, M., Dowling, RJ, Sonenberg, N., and Pollak, MN 2008. The effects of adiponectin and metformin on prostate and colon neoplasia involve activation of AMP-activated protein kinase. Cancer Prev Res (Phila) 1: 369-375.

45. Buzzai, M., Jones, RG, Amaravadi, RK, Lum, JJ, DeBerardinis, RJ, Zhao, F., Viollet, B., and Thompson, CB 2007. Systemic treatment with the antidiabetic drug metformin selectively impairs p53- deficient tumor cell growth. Cancer Res 67: 6745-6752.

46. Ben Sahra, I., Laurent, K., Loubat, A., Giorgetti-Peraldi, S., Colosetti, P., Auberger, P., Tanti, JF, Le Marchand-Brustel, Y., and Bost, F. 2008. The antidiabetic drug metformin exerts an antitumor effect in vitro and in vivo through a decrease in cyclin D1 level. Oncogene 27: 3576-3586. doi: 3510.1038 / sj.onc.1211024. Epub 1212008 Jan 1211021.

47. Huang, X., Wullschleger, S., Shpiro, N., McGuire, VA, Sakamoto, K., Woods, YL, McBurnie, W., Fleming, S., and Alessi, DR 2008. Important role of the LKB1-AMPK pathway in suppressing tumorigenesis in PTEN-deficient mice. Biochem J 412: 211-221.

48. Bolen, S., Feldman, L., Vassy, ​​J., Wilson, L., Yeh, HC, Marinopoulos, S., Wiley, C., Selvin, E., Wilson, R., Bass, EB, et al. 2007. Systematic review: comparative effectiveness and safety of oral medications for type 2 diabetes mellitus. Ann Intern Med 147: 386-399.

49. Wulffele, MG, Kooy, A., Lehert, P., Bets, D., Ogterop, JC, Borger van der Burg, B., Donker, AJ, and Stehouwer, CD 2003. Effects of short-term treatment with metformin on serum concentrations of homocysteine, folate and vitamin B12 in type 2 diabetes mellitus: a randomized, placebo-controlled trial. J Intern Med 254: 455-463.

50. Andres, E., Noel, E., and Goichot, B. 2002. Metformin-associated vitamin B12 deficiency. Arch Intern Med 162: 2251-2252.

51. Gilligan, MA 2002. Metformin and vitamin B12 deficiency. Arch Intern Med 162: 484-485.

52. Ting, RZ, Szeto, CC, Chan, MH, Ma, KK, and Chow, KM 2006. Risk factors of vitamin B (12) deficiency in patients receiving metformin. Arch Intern Med 166: 1975-1979.

53. Salpeter, SR, Greyber, E., Pasternak, GA, and Salpeter, EE 2003. Risk of fatal and nonfatal lactic acidosis with metformin use in type 2 diabetes mellitus: systematic review and meta-analysis. Arch Intern Med 163: 2594-2602.

54. Pommier, Y. 2006. Topoisomerase I inhibitors: camptothecins and beyond. Nat Rev Cancer. 6: 789-802.

55. Saltz, LB, Cox, JV, Blanke, C., Rosen, LS, Fehrenbacher, L., Moore, MJ, Maroun, JA, Ackland, SP, Locker, PK, Pirotta, N., et al. 2000. Irinotecan plus fluorouracil and leucovorin for metastatic colorectal cancer. Irinotecan Study Group. N Engl J Med. 343: 905-914.

56. Liu, LF 1989. DNA topoisomerase poisons as antitumor drugs. Annu Rev Biochem. 58: 351-375.

57. Shao, RG, Cao, CX, Zhang, H., Kohn, KW, Wold, MS, and Pommier, Y. 1999. Replication-mediated DNA damage by camptothecin induces phosphorylation of RPA by DNA-dependent protein kinase and dissociates RPA : DNA-PK complexes. Embo J. 18: 1397-1406.

58. Morris, EJ, and Geller, HM 1996. Induction of neuronal apoptosis by camptothecin, an inhibitor of DNA topoisomerase-I: evidence for cell cycleindependent toxicity. J Cell Biol. 134: 757-770.

59. Loe, DW, Deeley, RG, and Cole, SP 1996. Biology of the multidrug resistance-associated protein, MRP. Eur J Cancer. 32A: 945-957.

60. Chen, ZS, Furukawa, T., Sumizawa, T., Ono, K., Ueda, K., Seto, K., and Akiyama, SI 1999. ATP-Dependent efflux of CPT-11 and SN-38 by multidrug resistance protein (MRP) and its inhibition by PAK-104P. Mol Pharmacol. 55: 921-928.

61. Chu, XY, Suzuki, H., Ueda, K., Kato, Y., Akiyama, S., and Sugiyama, Y. 1999. Active efflux of CPT-11 and its metabolites in human KB-derived cell lines. J Pharmacol Exp Ther. 288: 735-741.

62. Reid, RJ, Benedetti, P., and Bjornsti, MA 1998. Yeast as a model organism for studying the actions of DNA topoisomerase-targeted drugs. Biochim Biophys Acta. 1400: 289-300.

63. Kanzawa, F., Sugimoto, Y., Minato, K., Kasahara, K., Bungo, M., Nakagawa, K., Fujiwara, Y., Liu, LF, and Saijo, N. 1990. Establishment of a camptothecin analogue (CPT-11) -resistant cell line of human non-small cell lung cancer: characterization and mechanism of resistance. Cancer Res. 50: 5919-5924.

64. Giovanella, BC, Stehlin, JS, Wall, ME, Wani, MC, Nicholas, AW, Liu, LF, Silber, R., and Potmesil, M. 1989. DNA topoisomerase I - targeted chemotherapy of human colon cancer in xenografts. Science 246: 1046-1048.

65. Husain, I., Mohler, JL, Seigler, HF, and Besterman, JM 1994. Elevation of topoisomerase I messenger RNA, protein, and catalytic activity in human tumors: demonstration of tumor-type specificity and implications for cancer chemotherapy. Cancer Res. 54: 539-546.

66. Rubin, EH, Li, TK, Duann, P., and Liu, LF 1996. Cellular resistance to topoisomerase poisons. Cancer Treat Res. 87: 243-260.

67. Saleem, A., Edwards, TK, Rasheed, Z., and Rubin, EH 2000. Mechanisms of resistance to camptothecins. Ann NY Acad Sci. 922: 46-55.

68. Fisher, DE 1994. Apoptosis in cancer therapy: crossing the threshold. Cell 78: 539-542.

69. Wang, CY, Mayo, MW, and Baldwin, AS, Jr. 1996. TNF- and cancer therapy-induced apoptosis: potentiation by inhibition of NF-kappaB. Science 274: 784-787.

70. Cusack, JC, Jr., Liu, R., and Baldwin, AS, Jr. 2000. Inducible chemoresistance to 7-ethyl-10- [4- (1-piperidino) -1-piperidino] carbonyloxycamptothe cin (CPT- 11) in colorectal cancer cells and a xenograft model is overcome by inhibition of nuclear factor-kappaB activation. Cancer Res. 60: 2323-2330.

71. Koizumi, N., Hatano, E., Nitta, T., Tada, M., Harada, N., Taura, K., Ikai, I., and Shimahara, Y. 2005. Blocking of PI3K / Akt pathway apoptosis enhancements induced by SN-38, an active form of CPT-11, in human hepatoma cells. Int J Oncol. 26: 1301-1306.

72. Eisenhauer, EA, Therasse, P., Bogaerts, J., Schwartz, LH, Sargent, D., Ford, R., Dancey, J., Arbuck, S., Gwyther, S., Mooney, M., et al. 2009. New response evaluation criteria in solid tumors: revised RECIST guideline (version 1.1). Eur J Cancer 45: 228-247.

73. National Cancer Institute, NIH, and DHHS. 2009. Common Terminology Criteria for Adverse Events v4.0.

74. Karapetis, CS, Khambata-Ford, S., Jonker, DJ, O'Callaghan, CJ, Tu, D., Tebbutt, NC, Simes, RJ, Chalchal, H., Shapiro, JD, Robitaille, S., et al. 2008. K-ras mutations and benefit from cetuximab in advanced colorectal cancer. N Engl J Med. 359: 1757-1765. doi: 1710.1056 / NEJMoa0804385.

75. Fuchs, CS, Moore, MR, Harker, G., Villa, L., Rinaldi, D., and Hecht, JR 2003. Phase III comparison of two irinotecan dosing regimens in second-line therapy of metastatic colorectal cancer. J Clin Oncol 21: 807-814.

76. Simon, R. 1989. Optimal two-stage designs for phase II clinical trials. Control Clin Trials 10: 1-10.

77. Zhou, K., Bellenguez, C., Spencer, CC, Bennett, AJ, Coleman, RL, Tavendale, R. Hawley, SA, Donnelly, LA, Schofield, C., Groves, CJ, et al. 2011. Common variants near ATM are associated with glycemic response to metformin in type 2 diabetes. Nat Genet 43: 117-120.

78. Scagliotti, GV, Krzakowski, M., Szczesna, A., Strausz, J., Makhson, A., Reck, M., Wierzbicki, RF, Albert, I., Thomas, M., Miziara, JE, et al. 2012. Sunitinib plus erlotinib versus placebo plus erlotinib in patients with previously treated non-small cell lung cancer: a phase III trial. J Clin Oncol 30: 20702078.

79. Tannock, IF, Fizazi, K., Ivanov, S., Karlsson, CT, Flechon, A., Skoneczna, I., Orlandi, F., Gravis, G., Matveev, V., Bavbek, S., et al. 2013. Aflibercept versus placebo in combination with docetaxel and prednisone for treatment of men with metastatic castration-resistant prostate cancer (VENICE): a phase 3, doubleblind randomized trial. Lancet Oncol 14: 760-768.

80. Tabernero, J., Dos Santos, LV, Tomasek, J., Aprile, G., Ferry, D., Cho, J., Melichar, B., Tehfe, M., Topuzov, E., Zalcberg, J. , et al. 2013. Phase 3, radomized trials of ramucirumab in patients with gastric metastatic or GEJ adenocarcinoma following progression on first-line chemotherapy. Ann Oncol 24: v14-iv14.

22. Appendix 1 Eastern Cooperative Oncology Group (ECOG)

Status Performance Scale

| Scale | description |
| --- | --- |
| 0 | Fully active; able to perform all your activities without restriction (Karnofsky 90-100%) |
| 1 | Restriction to rigorous physical activity; capable of light work and sedentary nature (Karnofsky 70-80%) |
| 2 | Able to perform all self-care but unable to perform any work activity; standing approximately 50% of the hours the patient is awake. (Karnofsky 50-60%). |
| 3 | Able to perform only limited self-care, confined to bed or chair more than 50% of waking hours (Karnofsky 30-40%) |
| 4 | Completely unable to perform basic self-care, fully confined to bed or chair (Karnofsky <30%). |
| 5th | death |

23. Appendix 2 - Definition of tumor evaluation by Response Evaluation Criteria in Solid Tumor (RECIST)

Measurable disease . Measurable lesions are defined as those that can be accurately measured in at least one dimension (longest diameter to be recorded) as > 20 mm with conventional techniques (physical examination, CT, MRI) or as > 10 mm with spiral CT. All tumor measurements should be recorded in millimeters (or decimal fractions of centimeters).

The same assessment method and technique should be used to characterize each injury identified and reported at baseline and during follow-up.

Clinical lesions : Clinical lesions will only be considered measurable when they are superficial (eg, skin nodules, palpable lymph nodes). For skin lesions, documentation by color photography, including a ruler for estimating the size of the lesion, is recommended.

  CT / MRI: CT and MRI should be the best reproducible and currently available methods for measuring target lesions selected for response assessment. Conventional CT and MRI should be made with cuts of 10 mm or less in cross section, contiguously. A spiral CT must be performed using a 5mm contiguous reconstruction algorithm. This applies to the chest, abdomen and pelvis.

Unmeasurable disease . All other lesions (or disease sites), including small lesions (diameter larger than <20 mm with conventional techniques or <10 mm with spiral CT) are considered non-measurable diseases. Bone lesions, leptomeningeal disease, ascites, pleural / pericardial effusions, lymphagitis, and cystic lesions are all not measurable.

  Target Injuries . All lesions measurable to a maximum of two organ lesions and five lesions in the total representation of all organs involved should be identified as target lesions and recorded and measured at baseline. Target lesions should be selected based on their size (lesions with the longest diameters) and their suitability for accurate repeated measurements (by imaging techniques or clinically). The sum of the largest diameter (LD) of all target lesions will be calculated and reported as the sum of LD at baseline. The baseline LD sum will be used as a reference and then characterize the objective tumor response of the measurable disease dimension. If there are> 5 measurable lesions, those not selected as target lesions will be considered, together with non-measurable diseases, as non-target lesions.

Non-target injuries . All non-measurable lesions (or disease sites) in addition to any measurable lesions and above 10 listed as target lesions. Measurements are not required, but these lesions should be observed at baseline and should be accompanied as “present” or “absent”.

All patients will have their BEST ANSWER in the study ranked as summarized below:

weeks

Partial Response (PR): a reduction of at least 30% in the sum of LD from target lesions by reference to the baseline LD sum and subsequent confirmation at ≥ 4 weeks.

Stable Disease (DS): steady state of the disease. Neither sufficient reduction to qualify as PR nor sufficient increase to qualify as PD, documented at least once > 4 weeks from baseline.

Progressive Disease (PD): an increase of at least 20% in the sum of LD of the measured lesions with reference to the lowest LD sum recorded since the beginning of treatment. The appearance of new lesions will also constitute progressive disease. Under exceptional circumstances, evident progression of non-target lesions may be accepted as evidence of disease progression.

Criteria for radiological response assessment

| Target Injuries | Non Target Injuries | New Injuries | answer  General | The best answer for this category also requires |
| --- | --- | --- | --- | --- |
| CR | CR | Not | CR | > 4 weeks confirmation |
| CR | Non-CR / Non-PD | Not | PR | > 4 weeks confirmation |
| PR | Non-PD | Not | PR |  |
| SD | Non-PD | Not | SD | Documented at least once > 4 weeks from baseline |
| PD | Any | Yes or no | PD | Not before SD, PR or CR |
| Any | PD * | Yes or no | PD |  |
| Any | Any | Yes | PD |  |
| * In exceptional circumstances, evident progression of non-target lesions may be accepted as evidence of disease progression.  Note :  Patients with a general deterioration of health require discontinuation of treatment without objective evidence of disease progression which at that time should be reported as "symptomatic deterioration". Every effort should be made to document objective progression even after treatment discontinuation. | | | | |

**B- Progression Free Survival and Overall Survival according to irinotecan refractoriness**

**Progression Free Survival**


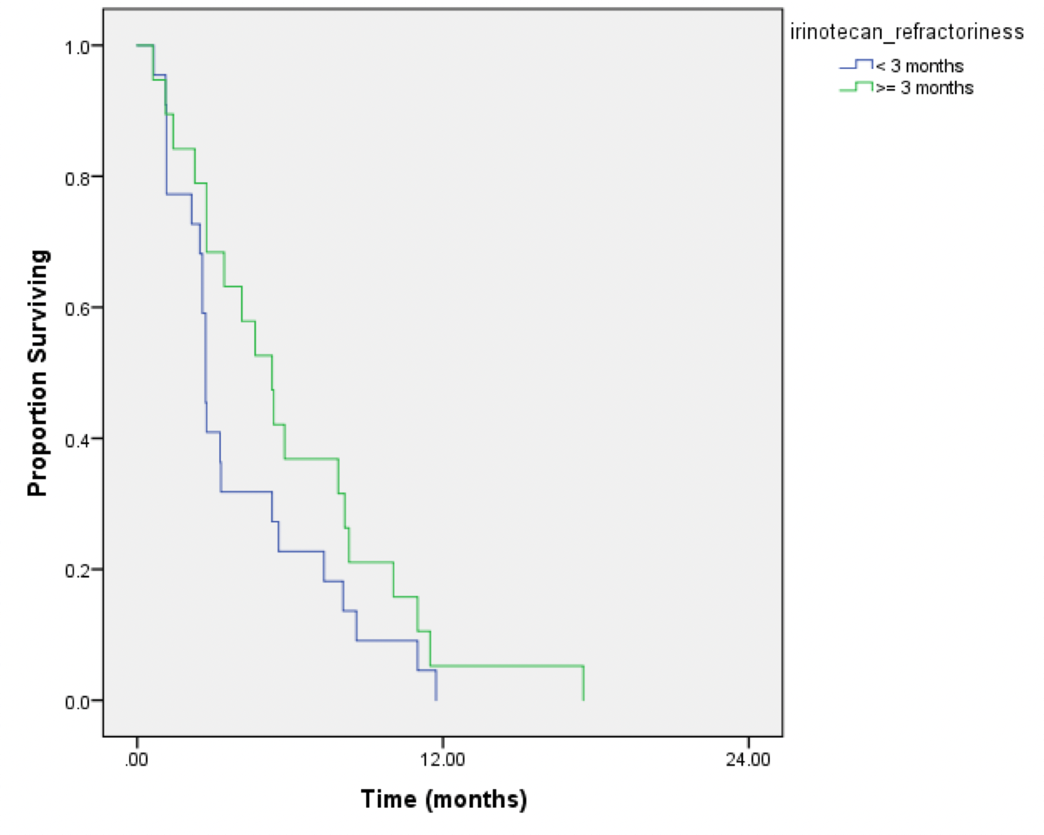


**Overall survival**


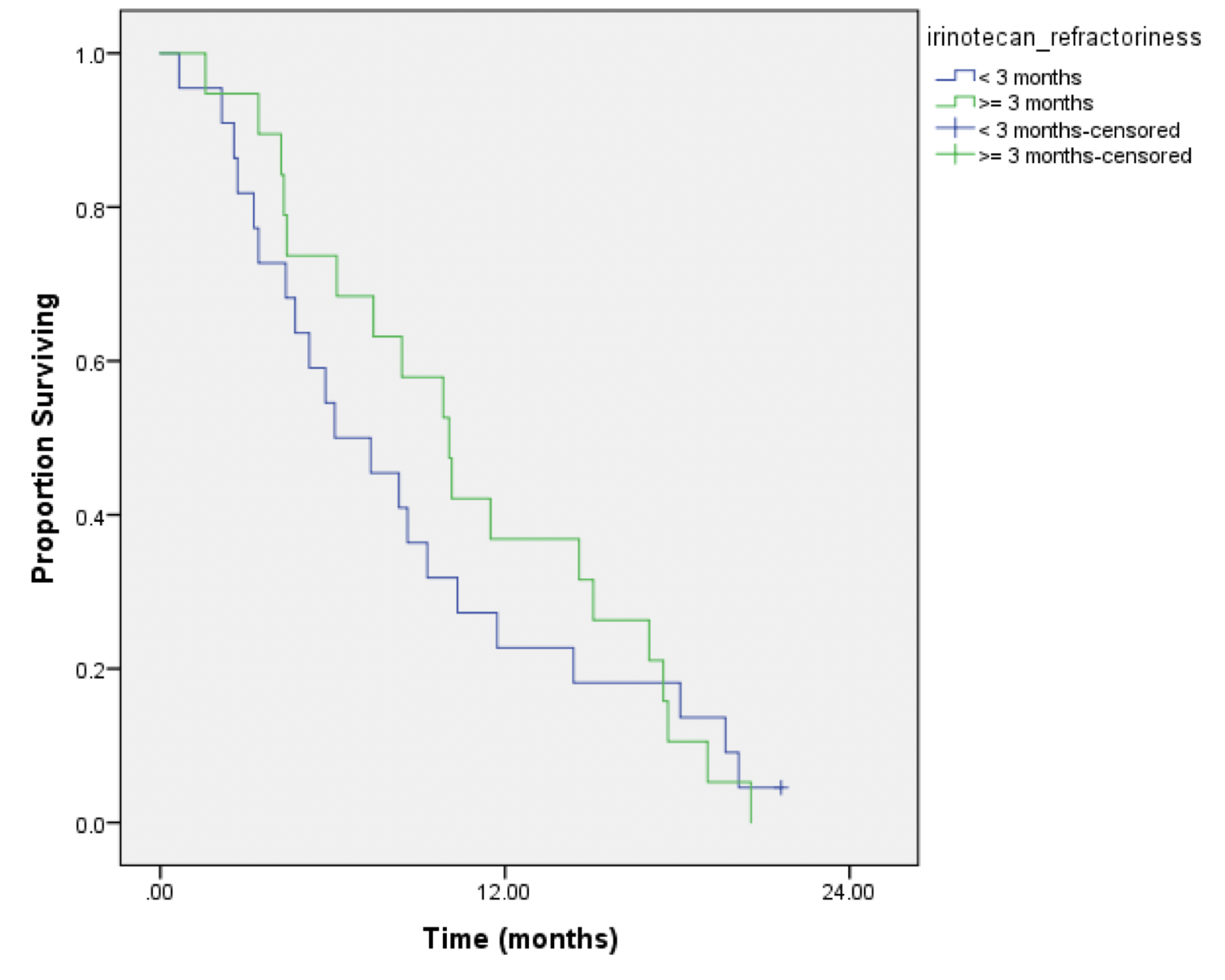

Supplement: Supplementary file 1 — Supplement 1 [file 41416_2020_1208_MOESM1_ESM.docx]
